# Radiotherapy remodels the tumor microenvironment for enhancing immunotherapeutic sensitivity

**DOI:** 10.1038/s41419-023-06211-2

**Published:** 2023-10-13

**Authors:** Senbo Liu, Wenkang Wang, Shengyun Hu, Bin Jia, Baojing Tuo, Haifeng Sun, Qiming Wang, Yang Liu, Zhenqiang Sun

**Affiliations:** 1 Department of Colorectal Surgery, The First Affiliated Hospital of Zhengzhou University, 450052 Zhengzhou, Henan China; 2 Henan Institute of Interconnected Intelligent Health Management, The First Affiliated Hospital of Zhengzhou University, 450052 Zhengzhou, Henan China; 3 Department of Breast Surgery, The First Affiliated Hospital of Zhengzhou University, 450052 Zhengzhou, Henan China; 4 Department of Oncology, The First Affiliated Hospital of Zhengzhou University, 450052 Zhengzhou, Henan China; 5 Department of Internal Medicine, The Affiliated Cancer Hospital of Zhengzhou University & Henan Cancer Hospital, 450001 Zhengzhou, China; 6 Department of Radiotherapy, The Affiliated Cancer Hospital of Zhengzhou University & Henan Cancer Hospital, 450001 Zhengzhou, China

**Keywords:** Cancer microenvironment, Cancer therapy, Tumour immunology

## Abstract

Cancer immunotherapy has transformed traditional treatments, with immune checkpoint blockade being particularly prominent. However, immunotherapy has minimal benefit for patients in most types of cancer and is largely ineffective in some cancers (such as pancreatic cancer and glioma). A synergistic anti-tumor response may be produced through the combined application with traditional tumor treatment methods. Radiotherapy (RT) not only kills tumor cells but also triggers the pro-inflammatory molecules’ release and immune cell infiltration, which remodel the tumor microenvironment (TME). Therefore, the combination of RT and immunotherapy is expected to achieve improved efficacy. In this review, we summarize the effects of RT on cellular components of the TME, including T cell receptor repertoires, different T cell subsets, metabolism, tumor-associated macrophages and other myeloid cells (dendritic cells, myeloid-derived suppressor cells, neutrophils and eosinophils). Meanwhile, non-cellular components such as lactate and extracellular vesicles are also elaborated. In addition, we discuss the impact of different RT modalities on tumor immunity and issues related to the clinical practice of combination therapy.

## Facts

- Cancer immunotherapy plays an important role in tumor treatment strategies.
- Objective tumor response rates and treatment sensitivity of cancer immunotherapy are still poor.
- The complicated and dynamic tumor microenvironment influences the efficacy of immunotherapy.
- Radiotherapy remodels the tumor microenvironment by affecting multiple cellular and non-cellular components.

## Open questions

- Can radiotherapy improve the efficacy of cancer immunotherapy by remodeling the tumor microenvironment?
- What are the specific mechanisms that radiotherapy remodels the tumor microenvironment?
- Does radiotherapy combined with immunotherapy have the potential for clinical application to help patients?

## Introduction

Cancer immunotherapy (CIT) is a treatment method that stimulates the immune system to attack and suppress tumor development, including promoting immune activation and relieving immune suppression [1]. This treatment has shown unprecedented response in patients and brought hope to many cancer patients. This strategy includes immune checkpoint blockade (ICB), adoptive cell therapy (ACT), cancer vaccines, and other approaches that target specific molecules or pathways involved in immune suppression or activation. Currently, the most widely studied and applied immunotherapy is ICB targeting immune checkpoint (IC) molecules such as programmed cell death 1 (PD-1), programmed cell death ligand 1 (PD-L1) and cytotoxic T lymphocyte-associated protein 4 (CTLA-4). Although immunotherapy has demonstrated significant efficacy in clinical applications, only a small proportion of patients benefit, with an objective response rate of 10–30% [2]. The implementation of CIT requires a thorough understanding of the interaction between the tumor and the immune system and the intricate regulatory networks in the tumor microenvironment (TME).

To maximize the therapeutic effect, the combination of immunotherapy with other therapeutic approaches has gradually gained attention. Radiotherapy (RT) induces micronuclei in tumor cells to activate cytoplasmic nucleic acid sensors, further activating the cyclic GMP-AMP synthase-stimulator of interferon genes (cGAS-STING) pathway and the expression of type I interferon (IFN-I) [3, 4]. The resulting inflammatory signaling effect remodels the TME. At the same time, RT triggers the expression and presentation of pre-existing and specific neoantigens in tumor cells, increasing immunogenicity [5]. Therefore, RT is early included in the category of combined immunotherapy research.

This review provides insight into radiation therapy’s immunomodulatory and remodeling effects on the TME, including immune cells and non-cellular components. Meanwhile, this review also includes exploring RT combined with immunotherapy as a potential cancer treatment strategy.

## Role of radiotherapy on T cells in the TME

T cells are the main component of lymphocytes and play an essential part in the immune response through cellular contacts or the killer cytokines’ secretion, serving as the body’s fighters against disease, infection and tumor formation. The roles of T cells in the TME and CIT are multiple [6]. This section focuses on the effects of RT in terms of T cell receptor (TCR) repertoires, cell clones, effector T cells, memory T cells, exhausted T cells, regulatory T cells (Tregs) and cell metabolism.

### T cell receptor repertoires and T cell clones

The TCR is a complex on the surface of T cells that recognizes antigens and mediates immune response, which has sensitivity and specificity to antigens presented by major histocompatibility complex (MHC) on antigen-presenting cells (APCs) [7]. The generation of different clonotypic TCRs is mainly attributed to rearrangement combinatorial diversity, linkage diversity and N sequence insertion or deletion of TCR genes [8]. The complementarity determining region 3 (CDR3) of the TCR β chain is unique to individual T cell clones, which is the key region for investigating TCR [9]. The TCR repertoire varies with the occurrence and progression of the disease, reflecting the changes the immune system undergoes to adapt to the environment. The diversity of TCRs allows the receptors to recognize different antigens, thereby stimulating an effective adaptive immune response [7].

Heterogeneity is one of the main barriers to tumor treatment, which is also characterized by differences in TCR repositories, including clonal compositions, clonotypes and CDR3 diversity [10]. The TCR repertoire’s diversity and T cell clones’ expansion are inextricably correlated with the efficacy and prognosis of CIT [11, 12]. The role of RT in this process is also attracting more attention. By analyzing the cumulative frequency of intratumoral clonotypes, the top 100 most abundant clonotypes in the RT arm represented 54% of the T cell repertoire, while the non-RT represented 43% of that. Analysis of CDR3 amino acid sequences revealed that dominant motifs accounted for a greater proportion of the intratumoral repertoire in the RT group [13]. Radiation therapy induced local expansion of intratumoral pre-existing T cell clones, accompanied by infiltration of unique clones within the irradiated tumor, which dominated the TCR repertoire [14]. In addition, RT augmented the diversity of the TCR repertoire of tumor-infiltrating lymphocytes (TILs), with an increase in TCR clonal diversity [15]. Consistently, lung cancer patients who responded to RT and CTLA-4 blockade had larger TIL-TCRs [5].

In parallel to intratumoral clones, T-cell clones in peripheral blood are also of interest. Increased expanded and contracted clones were observed in the peripheral blood of responders on day 22 after RT. Radiation therapy induced an upregulation of the tumor-derived KPNA2 gene expression (encoding karyopherin α2), a mutation recognized by the expanded clone [5]. Another study revealed that the expanded clonotypes in peripheral blood samples were largely tumor-enriched clonotypes, suggesting an overlap in the repertoire between tumor and blood samples after RT [13]. More importantly, the combination of RT and PD-1 blockade facilitated the translocation of expanded clones from irradiated tumor to unirradiated tumor and peripheral blood. This may unlock PD-1/PD-L1 axis-mediated adaptive resistance, thereby eliciting a broader polyclonal T-cell response [14]. In light of this, the overall expansion and contraction of peripheral blood T cell clones have the potential to determine when to introduce immunotherapy after RT. Given the accessibility of blood samples, this can also be utilized for monitoring the sensitivity of patients’ responses, thus predicting the effectiveness of combination therapy.

### T cell subsets

**Fig. 1:**
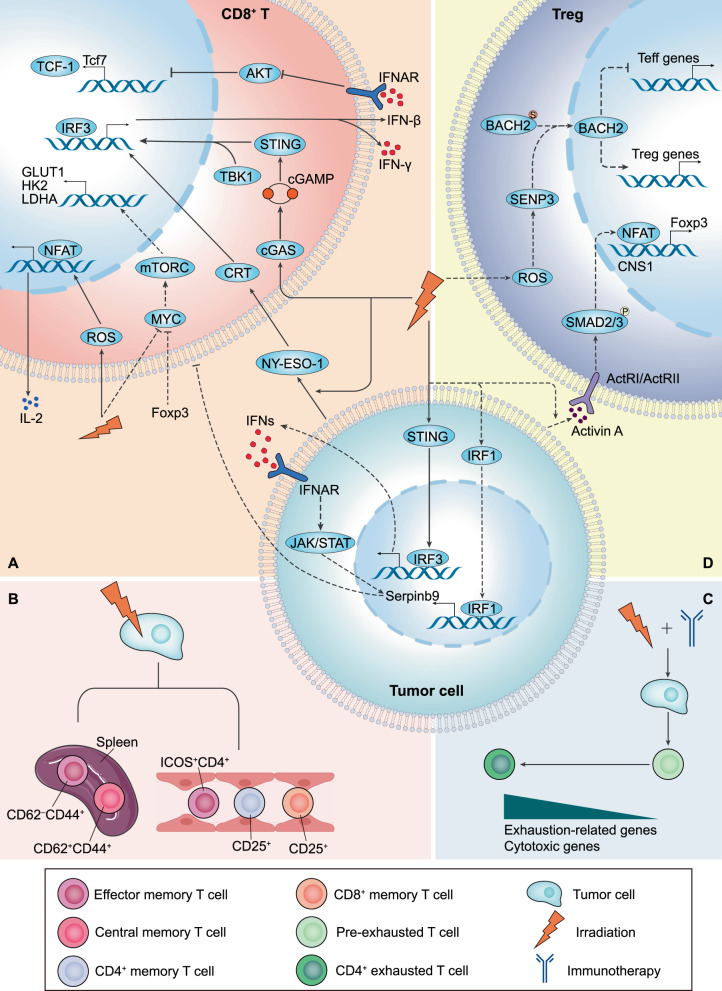
Irradiation-mediated alterations of T cells in the TME. **A** RT increases the expression of tumor-associated antigen NY-ESO-1, which binds CRT and thus activates CD8^+^ T cells to secrete more IFN-γ. Irradiation induces cytoplasmic DNA, which is sensed by cGAS, generating the second messenger cGAMP, thereby activating the STING protein. STING has been shown to induce IFN-β production by recruiting TBK1 to activate IRF3. IFN-β acts in an autocrine manner on the IFNAR of T cells, inhibiting AKT activity and promoting TCF-1 expression, which maintains the T cell stem cell-like state. However, RT activates IRF1 to promote Serpinb9 gene expression, thereby blocking CD8^+^ T cell attack. On the other hand, irradiation induces tumor cells to secrete IFNs, working on the IFNAR to activate Serpinb9 gene expression through the JAK/STAT pathway. In addition, irradiation reduces MYC expression levels and downregulates GLUT1, HK2 and LDHA genes involved in glucose uptake and glycolysis of T cells through mTORC regulation. Treg cell transcription factor Foxp3 reprograms cellular metabolism by repressing MYC. RT elicits an increase in ROS, activates NFAT and subsequently promotes IL-2 production, thereby activating effector T cells. **B** RT increases CD62^-^CD44^+^ effector memory T cells and CD62^+^CD44^+^ central memory T cells in the spleen. Irradiation augments activated CD25^+^CD8^+^ memory T cells, CD25^+^CD4^+^ memory T cells and ICOS^+^CD4^+^ effector memory T cells in peripheral blood. **C** RT combined with immunotherapy (αPD-1 and αCTLA-4) facilitates the differentiation of pre-exhausted Th1-like cells into intratumoral CD4^+^ Tex cells, during which exhaustion-related and cytotoxic genes are upregulated. **D** RT enhances the secretion of activin A from tumor cells. Activin A binds to the corresponding receptor ActRI/ActRII, activates the receptor kinase activity and phosphorylates the intracellular mediator SMAD2/3. SMAD2/3 translocates to the nucleus and binds to the CNS1 together with the NFAT. This promotes the transcription of Foxp3. Irradiation induces ROS production, which is reported to stabilize and accumulate SENP3, thereby mediating deSUMOylation of the transcription factor BACH2 and maintaining the immunosuppressive effects of Tregs. Solid lines represent anti-tumor effects and dashed lines represent pro-tumor effects.

T cells are present in different developmental stages or functional subsets during the body’s fight against pathogenic infections and tumor formation [16]. The effects of RT on different T subsets are also variable. Understanding these differences contributes to our comprehensive insight into the remodeling of T cells in the TME by RT. Here, we address cytotoxic T cells, memory T cells and exhausted T cells (Fig. 1).

The predominant role of cytotoxic or effector T cells is to secrete perforins and granzymes that kill infected and mutated cells [17]. They are also the “number one killer” of CIT. The main surface marker of cytotoxic T cells is CD8. Irradiation upregulated the expression of Fas on tumor cells, thereby boosting the killing capacity of effector T cells [18]. Significantly elevated CD3^+^CD8^+^Foxp3^-^ cytotoxic T cells were detected in tumor samples from glioma patients after RT [19]. Radiation therapy induced high levels of the tumor-associated antigen (TAA) NY-ESO-1 expression, which enhanced the expression of co-stimulatory molecules by binding to calreticulin (CRT), subsequently activating CD8^+^ T cells to produce more IFN-γ [20]. The highest infiltrating CD8^+^ T cells were identified at 1 Gy RT, localized in the intraepithelial tumor compartment. This depended on IFN signaling (including IFN-α and IFN-γ responses) [21]. However, IFN signaling was found to act in a dual manner. By regulating the IFN-inducible gene Serpinb9, IFN-I signaling protected tumor cells from attack from CD8^+^ T cells [22]. This sheds light on why RT both strengthens the anti-tumor immune response and mediates resistance. In addition, in the B16F10 mouse melanoma model, 10 Gy RT recruited a 7-fold higher area of cytotoxic CD8^+^ T cells than the control group and achieved the best tumor regression (around 80%). The immunohistochemical staining of blood vessels revealed a decrease in tumor vessel density, a thick wall and a visible layer of pericytes adhering to the surface, indicating normalization of the tumor vasculature [23]. Natural killer T (NKT) cells are a unique subpopulation of T cells with killing capacity, which co-express T cell and NK cell receptors [24]. NKT cells are activated by the specific glycolipid antigen α-galactosylceramide (α-GalCer) and proliferate and differentiate into two directions, IFN-γ-producing and IL-4-producing NKT cells, with the former anti-tumor and the latter pro-tumor [25]. Although there was no significant difference in the number of NKT cells after RT, α-GalCer-activated NKT cells differentiated toward anti-tumor direction and secreted more IFN-γ [26].

Memory T cells consist primarily of central memory (TCM) cells located in lymphoid tissue, effector memory (TEM) cells that circulate between peripheral blood and tissue, and tissue-resident memory cells that reside in peripheral tissue [27]. These cells can be maintained for a long time, even for a lifetime, and perform their immune protective function rapidly and efficiently upon restimulation with antigens. The mystery between memory T cells and CIT is gradually unraveled with growing research. Eomes^+^CD69^+^CD45RO^+^ memory T cells predicted the efficacy of patients to ICB [28]. Tumor-draining lymph nodes-derived tumor-specific memory T cells were identified as a subpopulation that truly responded to PD-1 blockade [29]. RT alone or combined with anti-PD-L1 increased CD62^-^CD44^+^ TEM in TILs and spleen and CD62^+^CD44^+^ TCM in spleen [30]. Ionizing irradiation of tumor sites showed a 2.5-fold elevation in the percentage of activated CD25^+^CD8^+^ memory T cells and a 2-fold increase in activated CD25^+^CD4^+^ memory T cells and ICOS^+^CD4^+^ TEM in peripheral blood. Unexpectedly, the Luminex analysis revealed a decrease in the chemokines CCL5 and CXCL10, which were essential for memory T cell homing, migration and activation [31]. The correlation mechanism between these two contradictory results is unclear. It may be attributed to a shortened half-life, increased degradation or reduced transcription and translation of these cytokines. There is still a need for further research.

In patients with chronic infections and cancer, T cells are continuously stimulated by antigens and inflammation, gradually losing effector function and memory characteristics, called T cell exhaustion (Tex). The process is characterized by loss of effector function, increased and sustained expression of inhibitory receptors, altered epigenetic and transcriptional profiles and modified metabolic patterns [32]. Tex were divided into four phases based on Ly108 (Slamf6) and CD69 as markers: exhaustion progenitors 1 (Tex^prog1^, Ly108^+^CD69^+^), exhaustion progenitors 2 (Tex^prog2^, Ly108^+^CD69^-^), exhaustion intermediate (Tex^int^, Ly108^-^CD69^-^), and exhaustion terminally (Tex^term^, Ly108^-^CD69^+^). Tex^int^ regains cytotoxic effector function, particularly reinforced after PD-L1 blockade, whereas Tex^term^ cannot, which is closely related to CIT [33]. Therefore, the implications of RT on Tex status are a topic worth exploring. 4 × 3 Gy RT increased the proportion of TCF-1^+^PD-1^+^CD8^+^ stem cell-like Tex at day 7. High levels of cGAS, STING and p-STING were detected during this process, suggesting that the cGAS/STING signaling pathway was required to maintain the Tex stem cell-like state after radiation [34]. In T cells, cGAS senses DNA and generates the second messenger cGAMP, which in turn activates the STING junction protein to form a dimer. The dimer induces IFN-β production by recruiting TBK1 to activate the transcription factor IRF3. IFN-β acts in an autocrine manner on the IFN receptor of T cells and inhibits Akt activity, thereby promoting the expression of TCF-1 and Slamf6 [35]. In addition, RT combined with immunotherapy (αPD-1 and αCTLA-4) was highly enriched with Tex and progenitor-exhausted T cells (Tpex). TCR sequencing identified CD4^+^ Tex and Tpex as the most clonally expanded cells, and CD4^+^ Tex may be differentiated from TEM, helper 1 T cell (Th1) or follicular helper (Tfh) cells. Subsequent pseudotime analysis revealed pre-exhausted Th1-like cells differentiated into intratumoral CD4^+^ Tex cells via an intermediate CD4^+^ Tpex state. In this process, exhaustion-related genes, cytotoxic genes and chemokine receptors were upregulated [21]. However, PD-L1 levels on tumor cells were raised, promoting T-cell exhaustion in post-RT-resistant melanoma. Encouragingly, the introduction of αPD-L1 reversed Tex, but αCTLA-4 did not [15]. αCTLA-4 mainly inhibits Tregs and may alleviate Tex theoretically. Therefore, the exact mechanism that αCTLA-4 is ineffective in reversing Tex remains to be explored.

In summary, the type of T cells characterizes their functional status, and various T cells play distinct roles throughout the course of tumor immunity. Effector T cells serve as the primary force for eliminating tumor cells. Memory T cells exhibit faster recognition and attack against cancer cells during recurrence. The reversal of Tex cells is crucial when T cells become dysfunctional during cancer progression and metastasis. Tailoring combination therapy to different stages of cancer, such as progression, metastasis, and recurrence, can leverage the unique advantages of diverse T cell types, aiding in the development of personalized treatment strategies.

### Regulatory T cells

Tregs specifically express forkhead box protein 3 (Foxp3), which plays a major role in their development and function. Depending on their origin, Tregs are divided into natural Tregs derived from the thymus (nTregs) and induced Tregs differentiated in the periphery (iTregs). Of these, nTregs account for the majority and mediate autoimmune tolerance. iTregs are generated by the conversion of peripheral naive T cells induced by transforming growth factor-β (TGF-β) or IFN-γ and negatively regulate anti-tumor immunity, with less stable Foxp3 expression [36].

Current findings on the effects of CIT on Tregs in the TME are variable. Administration of anti-PD-1/PD-L1 reverses the promotion of Tregs differentiation and proliferation by PD-L1, thereby impairing Tregs’ inhibitory capacity [37]. Conversely, blocking the interaction of PD-L1 with PD-1 and B7-1 by anti-PD-1/PD-L1 leads to augmented TCR and CD28 signaling in Tregs, which activates Tregs and potentiates suppressive function [38]. Similarly, the role of RT on Tregs in the TME is also complex. Accelerated local tumor irradiation decreased the frequency of Tregs, thus weakening the negative regulation on CD8^+^ T cells by the suppressive cytokines IL-10 and TGF-β, while conventional local tumor irradiation induced an increase in Treg levels. Treg depletion had a stronger contribution to tumor regression than the requirement of CD4^+^ T-cell help [39]. Although iTregs were more tolerant to 10 Gy irradiation and had lower cell death, Foxp3 expression was downregulated. They showed less ability to inhibit CD8^+^ T cell proliferation, likely attributed to the altered epigenetic status of the Foxp3 locus through partial methylation by radiation [40]. Furthermore, RT at 7.5 and 10 Gy decreased the proportion of Tregs in the spleen, while a single dose of 15 Gy increased [41]. Combined RT with immunotherapy (OX40/TLR agonist) resulted in an approximately 1.5-fold decrease in Treg density and Foxp3 expression levels in tumor tissue [42]. However, contradictory to these findings, 2 Gy RT raised the frequency of Tregs in tumors 2-fold higher than in the non-RT group. A significant elevation in Akt protein levels was detected in Tregs [43]. It has been reported that activated PI3K/Akt signaling upregulates the expression of anti-apoptotic factors such as Bcl2 and cellular inhibitors of apoptosis proteins-2, accompanied by a downregulation of caspase-3 to prevent apoptosis in CD4^+^ T cells [44]. The effect of Akt activation on Treg apoptosis remains to be further investigated. Irradiation increased tumor cell-derived activin A, which shares similar structures and SMAD2/3 signaling pathways with TGF-β. Combining RT with TGF-β blockade diminished Tregs in tumors with low baseline secretion of activin A, but boosted Tregs with high baseline. Triple therapy with the addition of immunotherapy (αCTLA-4 or αPD-1) prevented tumor recurrence in mice (80–100%) [45]. Activin A or TGF-β binds to the corresponding receptors (ActRI/ActRII or TGFβR) and activates receptor kinase activity, which phosphorylates the intracellular mediators SMAD2/3 [46]. SMAD2/3 translocates to the nucleus to bind the conserved noncoding sequences 1 (CNS1) together with the nuclear factor of activated T cells (NFAT), facilitating the transcription of Foxp3 [47] (Fig. 1). This mechanism provides new insights into how RT combined with immunotherapy eliminates the suppressive effect of Tregs.

### T cell metabolism

During tumorigenesis, T cells in the TME undergo metabolic reprogramming to obtain energy through aerobic glycolysis for maintaining T cell proliferation and activity [48]. Blocking T-cell glycolysis impairs their ability to produce IFN-γ, which is relevant to the efficacy of CIT [49]. Activation of TCR or CD28 promotes glucose transporter-1 (GLUT1) translocation to the cell membrane surface via the PI3K/Akt/mTORC1 pathway to increase glucose uptake, which in turn facilitates aerobic glycolysis [50]. However, PD-1 restrains glucose uptake and glycolysis and fosters lipid oxidation in T-cell activation. PD-1 blockade attenuates PI3K/Akt/mTORC1 signaling inhibition, thereby allowing T cells to revert to an effector cell-like metabolism [51]. Likewise, the role of RT in T-cell metabolic reprogramming should not be neglected (Fig. 1). Irradiation at 3 Gy decreased MYC mRNA expression levels and downregulated GLUT1, HK2 and LDHA genes involved in glucose uptake and glycolysis in T cells. Indeed, it was observed that T cells had lower glucose uptake and proliferated by less than 30% compared to over 85% of the control group [52]. Studies have reported that MYC and hypoxia inducible factor-1 (HIF-1) are involved in the metabolic reprogramming of activated T cells. When induced, they upregulate the expression of genes related to glycolytic enzymes, such as PKM1, HK2 and GLUT1 [48]. The high MYC-expressing cells differentiate into effector T cells, and the low differentiate into memory T cells, governed by mTORC1 [53]. In addition, RT causes an increase in reactive oxygen species (ROS), which facilitates the activation of effector T cells [54]. ROS activates NFAT and subsequently promotes IL-2 production [55]. However, ROS also induces Treg cell death leading to adenosine release, thereby suppressing T cell immunity [56]. Interestingly, ROS stabilizes and accumulates SUMO-specific protease 3 (SENP3) by blocking its ubiquitin-mediated degradation, which mediates the transcription factor BACH2 deSUMOylation (SUMOylation is an important reversible post-translational protein modification), thus maintaining the immunosuppressive activity of Tregs [57]. The transcription factor Foxp3 of Tregs reprograms cellular metabolism by inhibiting MYC, which impairs effector T cells by suppressing aerobic glycolysis [58]. The effects of cell metabolism on the TME and CIT are complicated and much effort is still required to delve into how RT shapes T cell metabolic reprogramming.

## Role of radiotherapy on tumor-associated macrophages in the TME

Tumor-associated macrophages (TAMs) are a critical component of the TME. Macrophages are involved in angiogenesis, extracellular matrix remodeling, cancer cell proliferation, metastasis and immunosuppression. On the other hand, macrophages, when properly activated, mediate phagocytosis and cytotoxic killing of cancer cells [59]. The plasticity of macrophages is evident in their ability to exhibit corresponding functional phenotypes in response to different states of the microenvironment, and these phenotypes can interconvert to adapt to environmental alterations. M1-type macrophages play an important role in killing pathogenic bacteria, tumor cells and anti-inflammatory responses. M2-type macrophages promote inflammation regression, tissue repair, immune escape and tumor progression [59, 60].

**Fig. 2:**
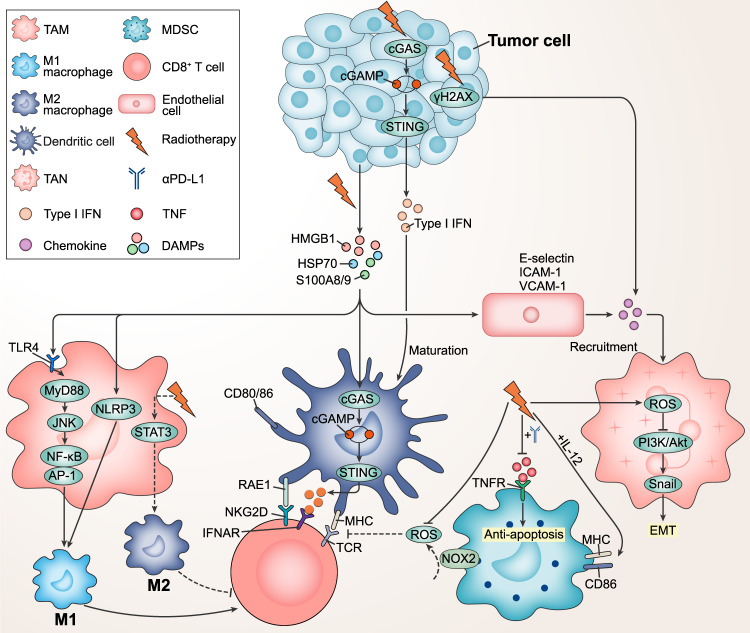
Effects of radiotherapy on myeloid cells in the TME. Irradiation induces an increase in HMGB1 release from tumor cells, activating TLR4 on TAMs. Activated TLR4 initiates NF-κB/AP-1 transcription factors via the MyD88/JNK signaling pathway, and triggers a pro-inflammatory-associated translational program that promotes M1-type polarization and stimulates CD8^+^ T cells. In addition, DAMPs activate NLRP3 inflammasome and launch pro-inflammatory genes, mediating M1 macrophage polarization. RT-induced DAMPs stimulate the expression of downstream type I IFNs through the cGAS/STING signaling pathway, thereby promoting the maturation of DCs (increased expression of CD80 and CD86). DCs present MHC to activate the TCR on CD8^+^ T cells. DCs also overexpress RAE1 after irradiation, which binds to NKG2D on CD8^+^ T cells. Moreover, RT-mediated elevation of DAMPs such as HMGB1, HSP70 and S100A8/9 results in increased expression of E-selectin, ICAM-1 and VCAM-1 on endothelial cells. Endothelial cells release chemokines such as IL-6, CXCL1, CXCL2 and CCL7 to recruit TANs. Irradiation increases the release of γH2AX, followed by elevated levels of chemokines CXCL1, CXCL2 and CCL5, which recruit TANs. RT induces high ROS production from TANs and inhibits PI3K/Akt phosphorylation, thereby reducing Snail expression to reverse the EMT. In addition, RT combined with αPD-L1 suppresses the TNF pathway, which has an anti-apoptotic function, thereby reducing MDSCs. RT combined with IL-12 treatment causes MDSCs to express higher MHC-II and CD86. MDSCs produce large amounts of ROS by the NOX2, which inhibits the formation of TCR and MHC antigen complexes in T cells. Irradiation inhibits the production of ROS in MDSCs to suppress this process. Solid lines represent anti-tumor effects and dashed lines represent pro-tumor effects.

Signals interfering with this plasticity vary considerably between tumors and even between different periods or sites of the same tumor, resulting in distinct TAM phenotypes. Tumor cell-derived cytokines (such as IL-6, IL-10, IFN-γ and TGF-β) and chemokines (such as CCL2, CCL5 and CXCL4) are key regulators of macrophage polarization [61, 62]. Local irradiation at 2 Gy induced CD11b^+^F4/80^+^Gr1^-^ TAMs to aggregate within the tumor rather than peritumorally. The increased iNOS^+^ macrophages (M1 type) suppressed the expression of Th2 cytokines (IL-4, IL-5, IL-6, IL-9 and IL-10) but recruited the Th1 chemokine CCL5, favoring the recruitment of T cells. Meanwhile, iNOS^+^ macrophages inhibited the expression of vascular endothelial growth factor and granulocyte-macrophage colony-stimulating factor (GM-CSF), contributing to vascular normalization [63]. These may be attributed to radiation activation of NOD-like receptor protein 3 (NLRP3) inflammasome, which initiates macrophage pro-inflammatory-related translational programs and mediates M1 macrophage polarization [64] (Fig. 2). In addition, the percentage of F4/80^+^iNOS^+^ macrophages was elevated 4-fold in primary and secondary tumors. High levels of high mobility group box 1 (HMGB1) and Toll-like receptors 4 (TLR4) were detected after irradiation [65]. Irradiation triggered the release of damage-associated molecular patterns (DAMPs) such as HMGB1 from tumor cells, thereby activating TLR4, which was associated with M1 macrophage polarization [66, 67]. Activated TLR4 was reported to upregulate chemokines expression via TLR4/MyD88 signaling and activate the JNK signaling pathway that led to NF-κB/AP-1 transcription factor initiation, thereby stimulating M1 polarization [68, 69] (Fig. 2). Consistently, RT also reduced intratumoral F4/80^+^CD206^+^ M2 macrophages by 1.5-fold [23]. However, CD163^+^ macrophages were observed to be increased after irradiation. Signal transducer and activator of transcription 3 (STAT3) activity (pSTAT3) and expression of cytokines IL-4, IL-5 and TGFβ1 were elevated, indicating M2 macrophage polarization [70] (Fig. 2). JAK2/STAT3 signaling has been reported to mediate M2 polarization and promote brain metastasis in non-small cell lung cancer [71]. In conclusion, RT favors M1 macrophage polarization and infiltration by invoking a pro-inflammatory environment and initiating immune-related pathways. INOS^+^ TAMs represent a population within the TME that can facilitate the efficacy of CIT. Inducing the expression of iNOS in TAMs offers a clinically applicable approach. Furthermore, adoptive transfer of iNOS-expressing macrophages may also hold promise as a prospective intervention method. Conversely, RT also promotes M2 polarization, which remains to be explored.

## Role of radiotherapy on other myeloid cells in the TME

In addition to TAMs, RT also reprogrammed other myeloid cells in the TME to modulate the immune response. Dendritic cells (DCs) are the most powerful APCs, delivering antigen-specific signals to T cells via MHC-peptide complexes bound to the TCR. DCs activate T cells through surface co-stimulatory molecules such as CD80, CD86 and CD40. They also secrete multifunctional pro-inflammatory cytokines such as IL-12, which stimulate the differentiation of naive T cells to effector T cells and activate NK cells [72]. DCs also make a significant contribution to immunotherapy against tumors. Studies in recent years have found that patients with high infiltration of DCs in solid tumors tend to achieve better prognosis [73]. Supernatants from irradiated tumor cells induced elevated expression of CD80 and CD86 on the surface of DCs and a 4-fold increase in infiltration of CD11b^+^CD11c^+^MHC-II^+^ DCs [74, 75]. A higher proportion of irradiated tumor cells were bound and phagocytosed by DCs than unirradiated tumor cells, and there was enhanced expression of TAA in the MHC [18]. The spatial distance between CD68^+^CD11c^+^ DCs and T cells was shortened, indicating the occurrence of cell-to-cell interactions [76]. RT induced an increased proportion of HMGB1^+^ tumor cells and upregulation of HMGB1 and STING protein expression in the TME, favoring the maturation of DCs [39, 77]. DAMPs (such as HMGB1) have been reported to activate the expression of downstream type I IFN via the cGAS/STING signaling pathway, thereby promoting the maturation of DCs [78]. DCs trafficked to tumors, took up tumor antigens and migrated to local lymph nodes where they cross-presented antigens to CD8^+^ T cells to mediate anti-tumor immunity [79] (Fig. 2). In addition, RT combined with immunotherapy reprogrammed cDC1 and cDC2/mono-like DC [21]. cDC1 expressed high levels of H2K1 and H2D1 (MHC-I molecules), suggesting heightened antigen presentation, and Batf3 was the key transcription factor driving this process. Enlargement of cDC2/mono-like DC enriched for MHC-I and II presenting, and IFN-I-related genes was observed. Consistently, infiltration of activated CD11b^+^CD11c^+^MHC-II^+^CCR2^+/-^ DCs triggered, and co-stimulatory ligands CD40, CD70, CD80 and CD86 expression augmented, suggesting maturation of DCs. More interestingly, a high frequency of cDC1 and cDC2 was noted, which overexpressed the NKG2D ligand RAE1, matching the elevated CD8^+^ and CD4^+^ TILs expressing NKG2D in tumors [21] (Fig. 2). These suggest that RT activates the maturation of DCs and thus triggers the ability of T cells to elicit anti-tumor responses. Radiation therapy, on the one hand, triggers the release of tumor antigens, and on the other hand, induces DAMPs, attracting immune cells into the tumor area. This provides a favorable environment for DC-based cell therapy (such as DC vaccines), which may enhance the clinical effectiveness of DC immunotherapy.

Myeloid-derived suppressor cells (MDSCs) develop from common myeloid progenitor located in the bone marrow and are divided morphologically into two subgroups: monocytic MDSCs (M-MDSCs) and granulocytic/polymorphonuclear MDSCs (G/PMN-MDSCs) [80]. They are minimally present in the peripheral blood of healthy humans but are greatly expanded in disease states such as inflammation or infection, especially following neoplasia. Subsequently, they migrate to the area of the lesion through peripheral blood circulation. MDSCs are attracted to the tumor area by a variety of cytokines and are subjected to extreme conditions of hypoxia, high oxidative stress and nutritional deficiency within the tumor [81]. Their function and differentiation undergo changes. For example, M-MDSCs migrate to the tumor site and differentiate into TAMs [82]. MDSCs function as suppressors of both acquired and innate immunity through multiple mechanisms, including blocking T-cell activation, disruption of activated T cells, inhibiting the cytotoxicity of NK cells and polarizing macrophages toward pro-tumor phenotype [83]. The level of MDSCs is closely related to the outcome of immunotherapy and the prognosis of the patient [84]. The positive effects of RT on MDSCs occurred mainly in the early stages. A decline in the proportion of intratumoral CD11b^+^Gr1^+^ MDSCs was detected 7 days after irradiation, but an increase in MDSCs at late tumor regrowth time points [75]. Moreover, RT fractionation protocols have an impact on the remodeling of MDSCs. At single irradiation, the proportion of M-MDSCs reduced with increasing irradiation dose, while that of G-MDSCs did not change significantly. In the dose-fractionation scheme (1.33 × 3 Gy), the proportion of M-MDSCs was elevated while G-MDSCs were reduced. Notably, the total cell abundance of G-MDSCs was lower, suggesting that the inhibitory capacity of total MDSCs was not reversed [85]. However, the combination of RT with immunotherapy has shown great potential. RT combined with αPD-L1 resulted in a diminishing of MDSCs in the TME, which was associated with the TNF or Fas/FasL pathways [86, 87] (Fig. 2). RT combined with IL-12 treatment decreased the percentage of intratumoral CD45^+^CD11b^+^Gr1^high^ MDSCs by 2-fold compared to the untreated group. RT/IL-12-treated MDSCs expressed higher levels of MHC-II and CD86 and exhibited attenuated suppression of T cell proliferation (T cell proliferation was reduced by 38–56% in the combined group and by over 90% in the untreated group) (Fig. 2). Furthermore, the percentage of ROS^+^ cells and the mean fluorescence intensity of ROS in MDSCs declined, suggesting the impaired suppressive capacity of MDSCs [88]. Activated MDSCs produce high levels of ROS in the presence of NADPH oxidase 2 (NOX2), which blocks the immune response by dampening the formation of TCR and MHC antigen complexes in T cells [89] (Fig. 2).

Neutrophils have a complex role in the TME and are described as tumor-associated neutrophils (TANs), classified as anti-tumor (N1) and pro-tumor (N2) phenotypes [90]. The pro-tumor mechanisms of neutrophils include promoting neovascularization, releasing neutrophil extracellular traps to enhance tumor cell proliferation, inducing extracellular matrix remodeling and immunosuppressive effects in response to TGF-β signaling. In the meantime, neutrophils release NO and H_2_O_2_ to kill tumor cells, increase the amount of IFN-γ in the TME to foster the anti-tumor effect of αβT cells and block the epithelial-mesenchymal transition (EMT) to exert a positive influence [91]. Reshaping of TANs by RT has been reported. The air pouch model revealed that supernatants from irradiated tumor cells recruited polymorphonuclear neutrophils. A rapid accumulation of Ly6G^+^ neutrophils was observed 6 h after injection of the supernatants by confocal immunofluorescence microscopy. DAMPs such as HMGB1, heat shock proteins (HSP) 70 and S100A8/9 were elevated in the TME, activating TLR4 and increasing expression of E-selectin, ICAM-1 and VCAM-1 on endothelial cells, thereby recruiting neutrophils. This activation pattern was characterized by the triggering and release of IL-6, CXCL1, CXCL2 and CCL7, which differed from the TNF activation pattern [92] (Fig. 2). In addition, neutrophil recruitment reached a peak 24 h after RT, which preceded the peak in cytotoxic T lymphocytes (CTLs). Irradiation caused the release of γH2AX (a marker of DNA damage), followed by elevated levels of chemokines CXCL1, CXCL2 and CCL5 to recruit TANs to the tumor site (Fig. 2). Ionizing irradiation mediated the release of large amounts of ROS from TANs and restrained PI3K/Akt phosphorylation, thereby reducing Snail expression. This reversed the EMT process, suggesting that neutrophils exerted an anti-tumor activity [93] (Fig. 2). More interestingly, myeloperoxidase (MPO) activity was boosted 10-fold in the TME after RT and the anti-tumor capacity of neutrophils was supported by high MPO activity. Traditionally MPO inhibitors have been used to treat tumors without RT, but this suggests that high MPO activity is required to maintain the anti-tumor response of TANs after RT [94].

Eosinophils also have a dual role and regulate tumor progression directly by interacting with tumor cells or indirectly by shaping the TME [95]. Eosinophils synthesize and release epidermal growth factor and TGFβ1 to induce tumor growth and EMT. Eosinophils trigger tumor cell death by secreting eosinophil cationic protein, eosinophil-derived neurotoxin and granzymes. Meanwhile, eosinophils recruit CD8^+^ T cells and exert cytotoxicity by releasing IFN-γ, CXCL9 and CXCL10 [95]. RT enhanced intratumoral eosinophil infiltration and raised the expression of genes involved in eosinophil differentiation, activation and chemotaxis. Transcription and translation of cytokines associated with eosinophil survival and proliferation (such as IL-5 and GM-CSF/CSF2) were also stimulated. Upregulated CCL11 and CCR3 in the TME drove the migration of eosinophils. Indeed, eosinophil infiltration augmented the anti-tumor efficacy of adoptive transferred T cell therapy, along with heightened CD8^+^ T cell infiltration and cytotoxicity [96]. Radiation-induced recruitment of intratumoral eosinophils is necessary for TME reprogramming to facilitate CTL-mediated anti-tumor immunity. RT remodeling of eosinophils has been relatively little studied, and the exact mechanisms need to be further explored.

## Effect of lactate on radiotherapy-mediated remodeling of the TME

Tumor cells obtain energy for growth and metabolism by converting glucose to lactate through glycolysis. To maintain metabolism, tumor cells excrete lactate via monocarboxylate transporters (MCTs), resulting in the acidic character of the TME [97]. Lactate accumulated in the TME has been shown to promote tumor progression. For instance, high concentration of lactate is transported into cells to be metabolized as a fuel substrate, and lactate supports tumor angiogenesis as well as tumor invasion and metastasis [98].

**Fig. 3:**
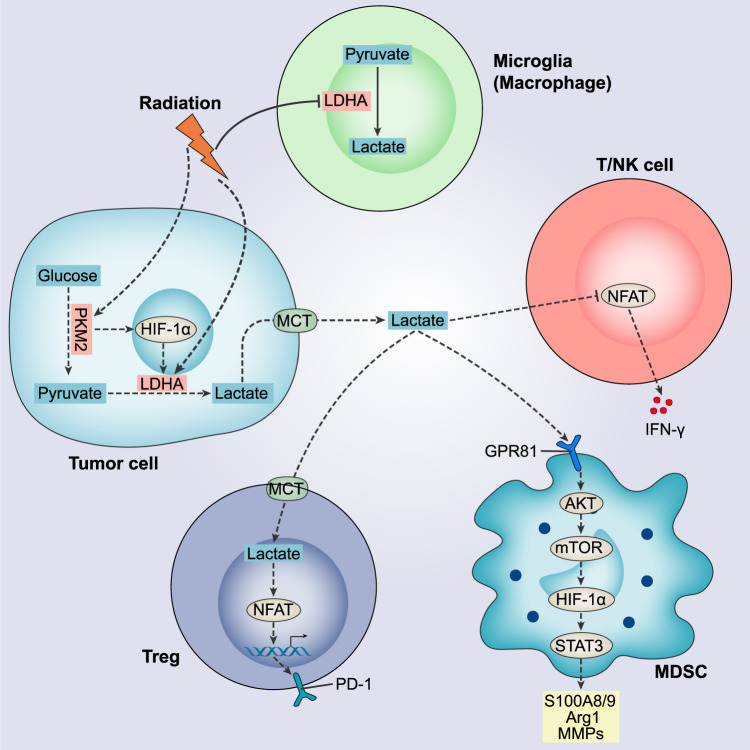
The value of lactate in the radiotherapy-mediated TME. RT upregulates the expression of PKM2 and LDHA in tumor cells, which catalyze glycolysis to produce lactate. Irradiation causes a significant increase in HIF-1α activity and regulates LDHA activity. PKM2 has been reported to stabilize HIF-1α. Lactate is transported into TME via MCT. Lactate accumulation inhibits NFAT in T and NK cells, thereby reducing IFN-γ synthesis. Lactate binds to GPR81 on MDSCs to activate Akt. Subsequently, functional genes S100A8/9, Arg1 and MMPs are upregulated through the mTOR/HIF-1α/STAT3 pathway, suggesting activation of MDSCs. Tregs take up lactate in TME via MCT1, which promotes NFAT1 entry into the nucleus and induces PD-1 expression. In addition, RT causes a decrease in LDHA expression and inhibits the conversion of pyruvate to lactate in microglia. Solid lines represent anti-tumor effects and dashed lines represent pro-tumor effects.

The glycolytic metabolism of cancer cells utilizes lactate dehydrogenase (LDH) to convert pyruvate to lactate. RT upregulated LDHA and PKM2 expression in pancreatic cancer cells and increased lactate for at least 120 h. HIF-1α activity was also significantly augmented, which regulates a series of glycolytic enzymes involved in the Warburg effect (Fig. 3). Subsequently, lactate was identified to bind to G protein-coupled receptor 81 (GPR81) on MDSCs, and elevated levels of phosphorylated Akt, mTOR, S6 and STAT3 were detected. Correspondingly, the expression of functional genes S100A8, S100A9, Arg1 and Mmps was enhanced in CD11b^+^Gr-1^+^ MDSCs, suggesting activation of the suppressive phenotype. High lactate levels mediated by RT stimulated MDSCs to cause radioresistance via the GPR81/mTOR/HIF-1α/STAT3 pathway [99] (Fig. 3). In addition, RT induced a decrease in microglia LDHA expression and pyruvate-to-lactate conversion rates [100]. Microglia are tissue-resident macrophages, thought to largely promote tumor formation and perform immunosuppressive function [101]. However, the role of RT-mediated lactate reduction in microglia on tumor growth needs further investigation. The high expression of LDHA in melanoma contributed to lactate accumulation, and the acidic environment dampened the activated NFAT expression in T and NK cells, thereby reducing IFN-γ synthesis and favoring tumor growth [102] (Fig. 3). Tregs efficiently took up lactate via MCT1 in the TME with abnormally elevated levels of glycolysis, promoting NFAT1 entry into the nucleus and inducing PD-1 expression on Tregs (Fig. 3). However, the expression of PD-1 on CD8^+^ T cells was inhibited by lactate, leading to a failure of PD-1 blockade [103]. Therefore, RT-induced alterations of lactate in the TME are closely correlated with immune cell status, which may be an attractive target for studying the mechanisms regulating the efficacy of RT and immunotherapy. It is noteworthy that, during or after radiation therapy, alleviating the lactate-mediated acidic microenvironment may not only overcome radiation resistance in clinical settings but also greatly aid in enhancing the benefits of immunotherapy.

## Role of extracellular vesicles in radiotherapy-mediated remodeling of the TME

Extracellular vesicles (EVs), including exosomes, microvesicles and apoptotic vesicles, mediate intercellular communication and carry proteins, nucleic acids, lipids and other bioactive molecules that modulate the behavior of receptor cells [104]. In the TME, EVs derived from cancer, immune and other non-immune host cells have different compositions and functions, leading to immune activation or suppression [105].

**Fig. 4:**
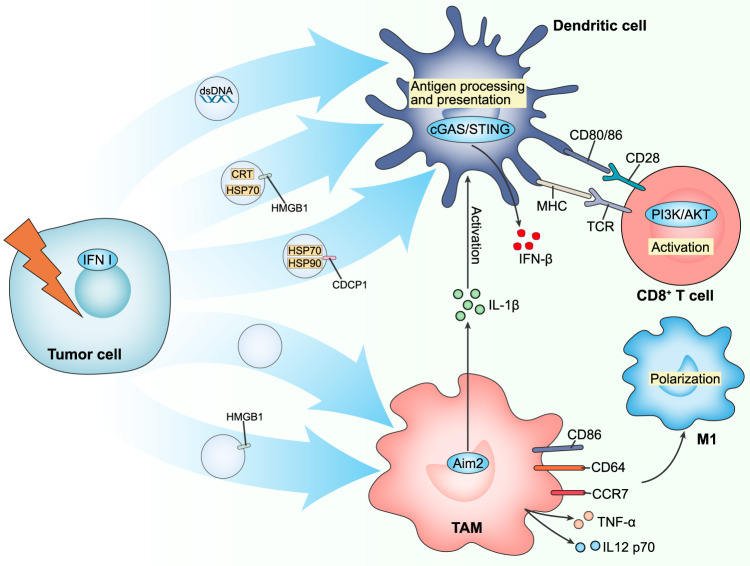
The value of EVs in the radiotherapy-mediated TME. The IFN-I pathway in tumor cells is activated by irradiation, and tumor cells release EVs carrying dsDNA. These EVs stimulate the expression of co-stimulatory molecules CD80 and CD86 and activate the cGAS/STING pathway in DCs to secrete IFN-β. RT promotes the release of exosomes from tumor cells to activate DCs, which contain HMGB1, CRT and HSP70. Similarly, irradiated tumors upregulate HSP70, HSP90 and a potential TAA, CDCP1 protein, in EVs. DCs have an enhanced ability to phagocytose EVs and perform antigen presentation, thereby activating CD8^+^ T cells via the PI3K/Akt signaling pathway. In addition, irradiated tumor cell-derived EVs activate Aim2 inflammasome in macrophages and induce IL-1β production to stimulate DCs. Contact of EVs carrying HMGB1 with TAMs results in increased expression of activation markers CD86, CD64, CCR7 and pro-inflammatory factors TNF-α and IL-12 p70, suggesting a conversion of M2 to M1 macrophages. Solid lines represent anti-tumor effects and dashed lines represent pro-tumor effects.

Tumor-derived EVs have been confirmed to promote tumor progression by polarizing M2-type macrophages, impairing the killing activity of NK cells and CTLs and impeding DCs differentiation and maturation. Conversely, they also carry TAAs, peptide-MHC and DAMPs to trigger antigen presentation and anti-tumor immunity [106]. Studies have found that irradiated tumor cell-derived EVs (TEVs) packed with DAMPs and tumor antigens (Fig. 4). Irradiation stimulated B16F10 melanoma cells to release exosomes enriched for the marker TSG101. HMGB1 was identified on the surface of exosomes, and CRT and HSP70 existed inside. Uptake experiments confirmed high levels of exosomes binding to CD11^+^ DCs and higher expression of co-stimulatory molecules CD40, CD80 and CD86, indicating activation of DCs. Intratumoral injection of irradiation-mediated exosomes augmented the number and percentage of intratumoral IFN-γ-producing NK cells to restrain tumor growth in a CD8^+^ T cell-independent manner [107]. Similarly, after 8 Gy irradiation, TEVs were collected and injected subcutaneously into each foot and eight spots on the back of each mouse, and enhanced intratumoral infiltration of CD4^+^ and CD8^+^ lymphocytes was observed. Proteomic analysis identified the upregulation of HSP70, HSP90 and a potential TAA, CDCP1, in TEVs, which activated DCs. The ability of DCs to phagocytose TEVs and perform antigen presentation was boosted, thereby motivating CD8^+^ T cells through the PI3K/Akt signaling pathway [108]. Exposure of 2 Gy irradiated TEVs to M2 macrophages resulted in elevated expression of activation markers CCR7, CD64, CD86 and pro-inflammatory cytokines TNF-α and IL-12 p70, implying M2 to M1 macrophage conversion. HMGB1 in TEVs was a potential signaling molecule to induce this process [109]. In addition, the IFN signaling pathway was one of the specific pathways in irradiated TEVs compared to untreated EVs, demonstrating the activation of the IFN-I pathway within cancer cells. TEVs carried dsDNA, regulated by the DNA exonuclease TREX1 in parental cells, inspired the co-stimulatory molecules CD40, CD80, CD86 expression and IFN-β secretion in DCs via the cGAS/STING pathway. TEVs inoculated in mice reached draining lymph nodes and interacted with DCs, thereby enhancing tumor-specific IFN-γ^+^CD8^+^ T infiltration [110]. More interestingly, irradiated TEVs activated AIM2 inflammasome and evoked IL-1β production and maturation. Activation of IL-1 signaling in DCs primed pre-existing CD8^+^ T cells and augmented anti-tumor immune responses in a cGAS-IFN-independent manner [111]. These findings also suggest that EVs carrying DAMPs can trigger the activation of DCs, NK and CD8^+^ T cells. This category of EVs presents a promising avenue in cancer treatment with potential clinical applications.

Immune cell-derived EVs also perform an essential regulatory function in the TME. TAMs-derived EVs maintained aerobic glycolysis of tumor cells via HIF-1α-stabilizing long noncoding RNA [112]. While NK cell-derived EVs contained the cytotoxic protein perforin and granzyme to induce tumor cell death [113]. Current research works on EVs in RT-mediated TME focus on those derived from tumor cells. Given the interest in immune cell-derived EVs, more insights regarding the role of irradiation-mediated immune cell-derived EVs in the TME are expected in the coming years.

## Effect of radiotherapy on immune checkpoint molecules in the TME

ICs are a class of immunosuppressive molecules that regulate the immune response. In the physiological state, ICs avoid tissue damage by regulating the persistence and intensity of the immune response. In contrast, tumor cells employ their immunosuppressive characteristics to evade the supervision of the immune system [114]. RT-induced changes in IC molecules correlate with tumor types, cell types, irradiation parameters and time points (Table 1).

**Table 1:** Modulation of IC molecules in the TME by radiotherapy.

IC molecules	Cell types	Changes	Cancer species	RT	Reference
PD-1	CD8^+^ T cells	↓	Pancreatic cancer	5 Gy × 5	[117]
	TAMs	↓	Rectal cancer	2 Gy	[109]
	CD4^+^ T cells CD8^+^ T cells	↑	Lung cancer	10 Gy × 4	[30]
	CD4^+^ T cells CD8^+^ T cells	↑	Gastric cancer	5 Gy × 3	[79]
	T cells	↑	Prostate cancer	6 Gy	[75]
PD-L1	Tumor cells	↑	Pancreatic cancer	5 Gy × 5	[117]
	Tumor cells	↑	Prostate cancer	6 Gy	[75]
	Tumor cells	↑	Colon cancer	8 Gy × 3	[120]
	Tumor cells	↑	Melanoma	2 Gy × 4	[118]
	Tumor cells	↑	Lung cancer	10 Gy × 4	[30]
				20 Gy	[15]
	Tumor cells	↑	Gastric cancer	5 Gy × 3	[79]
	Tumor cells	↑	Cervical squamous carcinoma	10 Gy	[121]
	Tumor cells	↑	Breast cancer	8 Gy × 3	[15]
				12 Gy	[86]
	TILs	↑	Melanoma	2 Gy × 4	[118]
	CD4^+^ T cells CD8^+^ T cells	↑	Lung cancer	10 Gy × 4	[30]
	CD8^+^ T cells	↑	HNSCC	10 Gy	[122]
	DCs	↑	Lung cancer	10 Gy × 4	[30]
	DCs	↑	Breast cancer	12 Gy	[86]
	MDSCs	↑	Lung cancer	10 Gy × 4	[30]
	Macrophages	↑	Breast cancer	12 Gy	[86]
CTLA-4	iTregs	↓	N/A	10 Gy	[40]
TIGIT	CD8^+^ T cells	↓	Colon cancer	2 Gy × 18	[120]
	CD3^+^CD4^+^ T cells CD3^+^CD8^+^ T cells	↓	OAC	2 Gy, 4 Gy	[127]
	CD8^+^ T cells	↑	Colon cancer	8 Gy × 3	[120]
TIM-3	CD3^+^CD4^+^ T cells CD3^+^CD8^+^ T cells	↓	OAC	2 Gy, 4 Gy	[127]
	NK cells	↑	Lung and liver cancer	Median: 10 Gy × 5	[31]
	CD8^+^ T cells	↑	HNSCC	10 Gy	[122]
	Tregs	↑	HNSCC	10 Gy	[122]
LAG-3	iTregs	↑	N/A	10 Gy	[40]

*IC* immune checkpoint, *RT* radiotherapy, *PD-1* programmed cell death 1, *PD-L1* programmed cell death ligand 1, *CTLA-4* cytotoxic T lymphocyte-associated protein 4, *TIGIT* T cell immunoreceptor with Ig and ITIM domains, *TIM-3* T cell immunoglobulin and mucin-containing molecule 3, *LAG-3* lymphocyte activation gene 3, *TAM* tumor-associated macrophage, *TIL* tumor-infiltrating lymphocyte, *DC* dendritic cell, *MDSC* myeloid-derived suppressor cell, *Treg* regulatory T cell, *iTreg* induced regulatory T cell, *NK* natural killer, *HNSCC* head and neck squamous cell carcinoma, *OAC* oesophageal adenocarcinoma, *N/A* not applicable.

PD-1 is an inhibitory receptor expressed on the surface of T or B cells, but also in NKT cells, DCs and monocytes, which plays a vital role in maintaining peripheral tolerance. PD-L1, the ligand of PD-1, is mainly expressed on the surface of APCs, while endothelial cells and epithelial cells also express PD-L1 [115]. High PD-L1 expression in tumor cells leads to sustained activation of the PD-1 pathway in the TME and suppression of T cell function to kill tumor cells. However, it has also been noted that high PD-L1 levels indicated that anti-tumor immunity was once activated and was associated with better clinical outcomes [116]. In pancreatic cancer, 5 Gy × 5 irradiation resulted in an increase in PD-L1 expression in tumor cells and a decrease in PD-1 in CD8^+^ T cells [117]. PD-L1 level of prostate cancer cells TRAMP-C1 was elevated at 72 h after 6 Gy and peaked at 24 h. PD-1 expression was raised in CD8^+^ T cells following tumor regrowth at 3 × 5 Gy, associated with tumor recurrence [75]. In 2 Gy × 4 fractionated RT-treated melanoma, PD-L1 increased on days 0 and 1 and decreased on day 3 in MEER tumors, whereas in B16F10 tumors PD-L1 increased continuously until day 3 and was also elevated in intratumoral TILs. PD-L1 upregulation in tumors by RT seemed to be mainly mediated by an enlarged number of myeloid cells with high PD-L1 expression [118]. RT-mediated upregulation of PD-L1 was reported to be achieved in a JAK/STAT1-dependent manner [119]. Similarly, elevated PD-L1 expression in tumor cells has been observed in lung, breast, gastric and colon cancers [15, 30, 79, 86, 120] (Table 1). In clinical samples of cervical squamous carcinoma treated with 10 Gy RT, PD-L1 expression in tumor cells was negatively correlated with the non-homologous end joining factor Ku80. This was confirmed in irradiation-treated Ku80-deficient HeLa cells, which may depend on ATR/Chk1 signaling [121]. Apart from tumor cells, regulation of PD-1 and PD-L1 was also found in immune cells after RT. After irradiation at 2 Gy, PD-1 expression was downregulated in TAMs, and phagocytic activity was augmented [109]. PD-1 and PD-L1 in CD4^+^/CD8^+^ T cells, DCs, macrophages and MDSCs were detected to be upregulated in different RT parameters and cancer species [30, 79, 86, 122] (Table 1).

CTLA-4 is a transmembrane receptor on T cells that competes with the co-stimulatory molecule CD28 for the ligand CD80/86, but has a substantially stronger affinity than the CD28. CTLA-4 inhibits T cell activation, APCs maturation and antigen-presenting ability and induces the proliferation of Tregs [115]. T cell immunoreceptor with Ig and ITIM domains (TIGIT) is expressed on CD4^+^/CD8^+^ T cells, NK cells and Tregs and represses immune cells at multiple steps of the cancer immunity cycle [123]. T cell immunoglobulin and mucin-containing molecule 3 (TIM-3) is expressed on the surface of CD4^+^/CD8^+^ T cells and interacts with galactin-9 ligand, mainly expressed on Tregs, to act as a T cell inhibitory receptor [124]. Lymphocyte activation gene 3 (LAG-3) is expressed on the surface of activated T, NK, B and pDC cells and exerts a negative regulatory effect on T cell proliferation by binding with high affinity to MHC-II [125]. Co-expression of LAG-3 and PD-1 by TILs was closely associated with their diminished function [126]. RNA sequencing and flow cytometry analysis showed that TIGIT of CD8^+^ T cells increased on day 7 after 8 Gy × 3 RT while 2 Gy × 18 irradiation treatment resulted in a drop in TIGIT expression over time (day 7, day 14 and day 30) [120]. In two cohorts of esophageal cancer, the expression of PD-1, PD-L1, TIGIT and TIM-3 was downregulated in CD3^+^CD4^+^ T and CD3^+^CD8^+^ T cells in patients well responding to 2 and 4 Gy RT while PD-1, PD-L1 and TIGIT were elevated in those poorly [127]. 10 Gy RT combined with αPD-L1 led to a 2- and 2.4-fold upsurge in the expression of TIM-3 in CD8^+^ T cells and Tregs, respectively, indicating upregulation of TIM-3 mediated resistance to PD-L1 blockade [122]. Moreover, CTLA-4 expression declined, and LAG-3 expression was enhanced by 10 Gy irradiation treatment of iTregs. The ability of iTregs to inhibit CD8^+^ T cell proliferation was attenuated due to decreased Foxp3 expression. CTLA-4, a regulatory gene of Foxp3, was associated with Foxp3 downregulation in Tregs [40]. More interestingly, immune infiltration in the TME was classified into high, medium and low categories based on the tumor inflammation signature (TIS). Upregulation of IC molecules PD-L1, CTLA-4, TIM-3 and glucocorticoid-induced tumor necrosis factor receptor was detected after RT. TIM-3 was higher in the high TIS region, while PD-1 and CTLA-4 were higher in the low TIS region, suggesting immunosuppression. However, the cellular origin of these molecules was not identified [76].

These findings propose a possible mechanism for the poor efficacy of RT combined with ICB and provide a rationale for combining multiple ICB. What kind of ICB is combined with RT depends on irradiation dose, cancer species and the infiltrating immune cells. Much work is still needed to dissect the exact mechanisms by which RT modulates IC molecules in the TME.

## Role of different radiotherapy modalities in remodeling the TME

RT is divided into conventional fractionated and hypofractionated therapy according to the single fractionated dose [128]. The former mostly uses a low dose of 1.8–2.0 Gy/fraction, 5 times/week regimen [129]. Conventional fractionated therapy is the most basic and commonly applied RT protocol. Hypofractionated RT increases the single dose and reduces the number of irradiations relative to conventional fractionation. The dose is usually more than 2.0 Gy/fraction, and typical modalities include stereotactic body radiation therapy (SBRT) and stereotactic ablative radiation therapy [130]. Different RT modalities have different effects on the remodeling of the TME and the tumor immune response.

Low-dose fractionated or unfractionated RT (dose ≤2 Gy) is found to be strongly associated with a pro-inflammatory environment and IFN signaling. 1 Gy irradiation upregulated the inflammation-related processes such as IFN-α and IFN-γ responses, complement activation and IL6/JAK/STAT3 signaling. The influx of T cells, NK cells and DCs was increased, and T cell influx was specific to tumor deposits, depending on IFN signaling. Low-dose RT (at 0.5 or 1 Gy per fraction, every two weeks, total dose 6 or 13 Gy, respectively) combined with ICB reprogrammed advanced immune-desert human tumors (gallbladder, ovarian and prostate cancer). Intratumoral Th1 signatures were significantly augmented, and innate and adaptive immune cells were recruited [21]. Consistently, whole-body irradiation at 0.075 Gy upregulated the expression of Th1-related genes Stat4, Socs1 and Sftpd and downregulated the negative regulator TGF-β by more than 4-fold in thymic lymphocytes. Low-dose irradiation induced Th1-type immune response, evidenced by a significant increase in IL-2 and IFN-γ secretion and enhanced toxic effects of killer T cells [131]. In addition, low-dose RT contributes to T-cell recruitment. 2 Gy irradiation induced vascular normalization and recruited tumor-specific T cells in a NO-dependent manner [63]. Five daily fractions of 2 Gy RT increased the frequency of IFN-γ expressing antigen-specific CD8^+^ T cells and elicited abscopal effect when combined with αPD-1 (>70% of mice with complete responses [CR]) [14]. Irradiation with 1 Gy × 2 fractions activated CD4^+^ and CD8^+^ T cells and suppressed TGF-β1 gene expression [132]. Moreover, low-dose RT also reprogrammed TAMs. 2 Gy irradiation triggered iNOS expression and downregulated the expression of M2-associated parameters HIF-1, Fizz-1, Ym-1 and arginase, reversing the Th2-dominant and tumor-promoting microenvironment initiated by TAMs [63].

Hypofractionated RT also recruits T cells and exhibits advantages in the induction of tumor antigens and activation of DCs. 15 Gy SBRT upregulated IL-16 gene (encoding T cell chemokine) and CXCL9, CXCL10, CXCL11 and the corresponding receptor CXCR3 expression, resulting in recruitment of T cells. Immunomodulatory interactions between lymphocytes and non-lymphocytes were the most significant pathways upregulated after RT [13]. SBRT attracted CD8^+^ T cell infiltration into the central tumor regions and markedly raised the ratio of central to marginal cells for 5 Gy × 5 consecutive days [133]. 8 Gy × 3 radiation boosted the release of the cytokines CXCL1, CXCL2 and CCL5 and early recruited neutrophils as first-line immune responders to generate anti-tumor function. Subsequently, the number of CTLs expanded and their activation status was strengthened [93]. SBRT also induced the expression of TAAs such as CA9, MUC1, 5T4 and NY-ESO1 and elevated the TP53 DNA damage response, which triggered immunogenic cell death (ICD) characterized by the release of HMGB1 and HSP70 [20, 117]. In addition, the supernatant of hypofractionated irradiated tumor cells elevated the percentage of migrating DCs and raised the activation markers CD80 and CD86 expression. The number of infiltrating intratumoral MHC-II^+^ APCs increased on days 5–10 after irradiation but decreased to the same level as the control on day 14 [74]. SBRT-treated TC-1 tumor cells co-cultured with DCs induced the highest percentage of CD11c^+^MHC-I^+^CD86^+^ mature DCs [134]. However, hypofractionated RT also invokes an immunosuppressive microenvironment. Immunosuppressive populations such as monocytes, macrophages and granulocytes persisted in the TME after SBRT. The ratio of suppressive myeloid cells to CD8^+^ T cells in the tumor nests (approximately 100:1) was slightly higher than in the untreated group, which may correlate with T cell anergy [117]. The proportion of immunosuppressive inflammatory monocytes, TAMs and TANs increased while MHC-II^+^ TAMs declined after SBRT, suggesting an impaired anti-tumor capacity [133]. SBRT at a dose of 6–10 Gy per fraction for 3–5 fractions contributed to a more than 2-fold increase in Foxp3^+^ Tregs infiltration density and a 1.5-fold increase in CD204^+^ macrophages [42]. These results suggest that the application of hypofractionated RT should be accompanied by consideration of the resistant effects of the immunosuppressive microenvironment. Strategies to counteract the negative environment may be necessary, such as combined immunotherapy to deplete suppressive cellular components.

## Effect of radiotherapy on TME in various cancer species

**Fig. 5:**
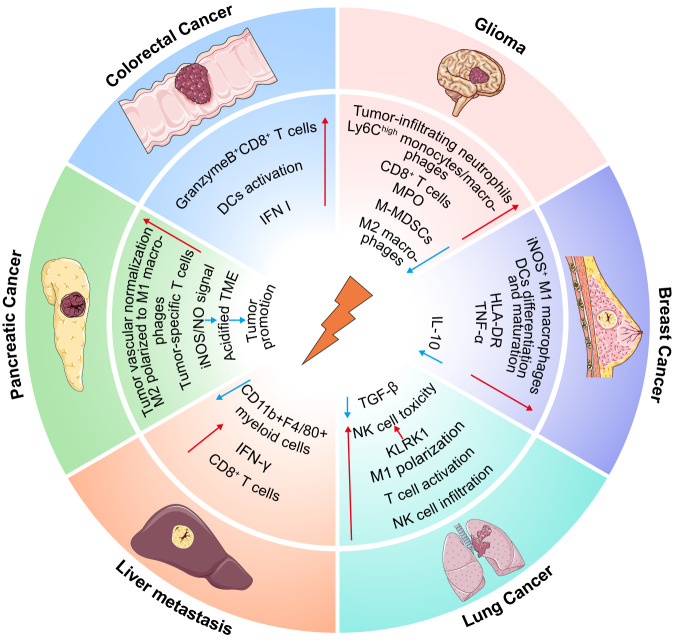
Radiotherapy remodels the TME in different cancer species. RT alters the TME in a variety of cancers, including tumor suppression and tumor promotion. RT induces differentiation and maturation of DCs, increases CD8^+^ T cells and effector molecules secretion, stimulates macrophage polarization and attenuates suppressive myeloid cells. However, RT facilitates tumor progression by acidifying the TME.

Different tumors have distinct cellular compositions and ratios in their TME, leading to varying immune responses that may explain the differences in sensitivity to immunotherapy. RT has been described previously to alter multiple immune cells and microenvironment components, serving as a basis to remodel the TME in different cancer types to improve immunotherapy’s efficacy (Fig. 5). Brain tumors have fewer tumor-infiltrating immune cells and are dominated by macrophages over lymphocytes and NK cells, which are resistant to ICB [135]. Glioma secreted factors to recruit and modify myeloid cells to create a microenvironment conducive to tumor growth and invasion. Irradiation resulted in a significant increase in tumor-infiltrating neutrophils and inflammatory Ly6C^high^ monocytes/macrophages and a 10-fold increase in MPO activity in the TME. In the irradiated environment, MPO shifted to the anti-tumor role, lowering the viability of glioma cells and diminishing their proliferation [94]. In addition, RT increased total T cells and CD8^+^ T cells in gliomas and reduced M2 TAMs/microglia and M-MDSCs by more than 5-fold [136]. The same results were found in another study [85]. In breast cancer, macrophage aggregation in tumors after RT (2.2- to 2.88-fold increase) was observed, with iNOS^+^ M1 macrophages rising from 14 to 73%. HMGB1 released from irradiated breast cancer cells stimulated M1-type macrophages to secrete high levels of TNF-α and low levels of IL-10, facilitating drastic anti-tumor activity [65]. RT fostered DC differentiation and maturation in the breast cancer microenvironment, as evidenced by augmented expression of the co-stimulatory ligands CD80 and CD86, the maturation marker CD83, the co-activator receptor CD40 and the MHC-II molecule HLA-DR [92]. Furthermore, RT boosted the anti-tumor immune response to lung adenocarcinoma through T cell activation, NK cell infiltration, M1 macrophage polarization and TGF-β reduction [132]. KLRK1, a representative gene of the NK cell receptor NKG2D, plays an important role in activating the NKG2D/NKG2D-Ls signaling pathway. As the clinical staging of lung cancer progressed, KLRK1 expression declined and NK cell activation status attenuated. Irradiation upregulated KLRK1 expression, and the most significant pathway was NK cell-mediated cytotoxicity by KEGG enrichment analysis. Irradiated NK cells exhibited elevated exposure levels of pro-inflammatory factors (IL-12, IL-18, IL-2 and CD16), and the infiltration of activated DCs, CD8^+^ T cells and cytotoxic cells increased [137].

Liver metastasis may inhibit CIT in a tumor-specific manner. Liver metastasis siphoned activated CD8^+^ T cells, and intratumoral CD11b^+^F4/80^+^ myeloid cells induced T cell apoptosis via the Fas-FasL pathway, thereby suppressing favorable immune responses. However, RT elicited increased CD8^+^ T cells, decreased CD11b^+^F4/80^+^ myeloid cells, augmented CD8^+^ and CD4^+^ cell proliferation and IFN-γ production in tumor-draining lymph nodes in situ, reshaping the immune microenvironment of liver metastasis [138]. Pancreatic cancer lesions have relatively high immune infiltration, but the predominant cells are macrophages [135]. RT shifted the M2-type effector cytokine milieu to M1-type in pancreatic cancer, as manifested by aggregation of iNOS-expressing cells and downregulation of M2-associated proteins such as Ym-1, Fizz-1 or arginase-1 [139]. RT also induced endothelial activation and Th1 chemokine expression and facilitated the normalization of tumor vasculature and differentiation of iNOS^+^ M1-type macrophages in pancreatic cancer. These allowed the effective recruitment of tumor-specific T cells [63]. However, conditioned medium from radiation-activated cancer-associated fibroblasts (CAFs) enhanced iNOS/NO signaling in tumor cells via NF-κB. This acidified the pancreatic TME to promote tumor growth. When iNOS inhibition was introduced, pancreatic tumor growth was retarded [140]. This suggests an important role of CAFs and iNOS signaling in the immunosuppressive microenvironment after RT for pancreatic cancer. For rectal cancer, T-cell inflammation gene expression profiling, IFN-I and macrophage populations were raised after RT [141]. Radiation therapy provoked a 5-fold increase in total T-cell accumulation in CT26 mice tumors, with a 7-fold increment in CD8^+^ T cells. The proportion of functional CD8^+^ T cells secreting granzyme B increased significantly from the first week and was maintained until 2 weeks after the end of irradiation [120]. In addition, irradiation upregulated the expression of activation markers CD80 and CD86 in DCs (1.5-fold) and increased MHC-II^+^ APCs by 3-fold in the colon cancer microenvironment. The titer of tumor-specific IgM antibodies was significantly higher (>2-fold) than that of controls, indicating that RT also had an effect on B cells [74].

## The value of radiotherapy combined with multiple immunotherapy

The ability of RT to remodel TME provides implications for combination with immunotherapy. Preclinical studies show that combining RT with various immunotherapies (e.g., ICB, ACT, cancer vaccines, cytokines, oncolytic viruses) can improve CIT efficacy. In addition, RT has an abscopal effect, which means that distant metastases beyond the irradiation range shrink or even fade away after localized tumor lesions receive RT [142]. Unfortunately, the abscopal effect after RT monotherapy is rarely observed in the clinical setting. RT combined with immunotherapy also has the potential to elicit the abscopal effect in preclinical models.

The administration of anti-PD-L1 boosted the response of post-radiation-resistant tumors. Anti-PD-L1/Anti-PD-1 combined with RT + anti-CTLA-4 yielded a significant survival benefit in mice, achieving an 80% CR rate. Moreover, after the introduction of anti-PD-L1/anti-PD-1, approximately three-fifths of mice remained alive for more than three months following tumor rechallenge. Notably, checkpoint blockade required RT to achieve high rates of complete remission [15]. Consistently, RT combined with anti-PD-L1 treatment effectively controlled the growth of breast tumors and MC38 colon cancer. At the evaluation endpoint, it was observed that IR + anti-PD-L1 reduced tumor volume by approximately 22- and 16-fold, respectively, compared to anti-PD-L1 alone. Subsequent tumor rechallenge in the combination treatment group revealed the absence of palpable tumors in mice, suggesting the generation of durable protective T-cell immunity. Further studies unveiled that the combination treatment also mediated the abscopal effect on contralateral tumors [86]. Anti-CTLA-4 administration alone had no effect on the growth of primary or secondary MCA38 tumors. Combination treatment significantly improved the growth inhibition of primary tumors (3:1 ratio of tumor weight in the anti-CTLA-4 alone to the combination group at day 35) and caused significant suppression of secondary tumors. An increase in the frequency of tumor-specific IFN-γ-expressing CD8^+^ T cells was detected in the combination group [143]. In addition, progress has been made in combining RT with ACT. Adoptive T cells alone resulted in regression of marginal EG7-OVA lymphoma. However, the combination of adoptive therapy and irradiation regressed tumors completely in all mice, and survival was significantly prolonged. Mice that completely rejected EG7-OVA tumors resisted the contralateral tumors in subsequent rechallenge, indicating the existence of a protective antigen-specific memory response [18]. In another study, a significant improvement in the survival of glioma mice was achieved when CAR-T cells were injected intravenously 1–3 h after irradiation compared to CAR-T cells only. At week 5, all mice in the single treatment group had died, while the combination treatment group had an 80% survival rate. Only in the combination group was an increase in CAR-T cells in the peripheral circulation detected at 3 weeks, confirming sustainable CAR-T cell activity [144].

RT also potentiates the efficacy of cancer vaccines. Mice receiving local irradiation combined with CTGF/E7 DNA vaccine exhibited significantly stronger therapeutic effects than the vaccine monotherapy. The combination group demonstrated more E7-specific IFN-γ-secreting CD8^+^ T cell precursors and infiltrating CD8^+^ cytotoxic lymphocytes [77]. Similarly, TC-1 cervical cancer mice first underwent local radiation at a single dose of 20 Gy, followed by a subcutaneous injection of PC7A nanovaccine and a booster immunization 7 days after the initial vaccination. The nanovaccine alone restrained slight tumor growth but not ultimate tumor growth. The combination group achieved 50% of mice tumor-free and additional growth inhibition in distal tumors 60 days after tumor inoculation [145]. Furthermore, it has also been shown that RT sensitizes cytokine therapy. IL-15 alone had no influence on the growth of mouse TSA breast tumors (poor immunogenicity), but significantly enhanced tumor response when combined with RT. 3 of the 11 mice treated with the combination therapy were tumor-free for >100 days, whereas all mice in the IL-15 alone group died by 30 days. Subsequent work revealed that batf3-dependent cDC1s were critical for initiating combination therapy-induced anti-tumor CD8^+^ T cell responses [146]. A modest reduction in pancreatic tumor burden was observed in the IL-12 alone group. The combination treatment group (24 h post-RT intratumoral injection of IL-12) eliminated tumors at day 20 post-implantation, with no detectable lesions until measurement termination by bioluminescence imaging. Long-term survival was achieved in 100% of mice. Combination treatment induced enhanced intratumoral T-cell activation and memory formation. The anti-tumor response was dependent on the full production of intratumoral IFN-γ. SBRT/IL-12 treatment resulted in the eradication of established liver metastases and a significant reduction in leg tumors (secondary lesions) (60% of mice were tumor-free at 25 days post-implantation), suggesting that the combination treatment drove abscopal effect [133]. In addition, oncolytic virus therapy selectively infects tumor cells causing acute tumor cell lysis and induces anti-tumor immunity [147]. SBRT combined with oncolytic virus achieved better tumor growth inhibition (approximately 3-fold) than virus monotherapy at day 39 after tumor inoculation in mice. None of the mice in the monotherapy group survived 80 days, whereas 40% of the mice in the combination group remained alive 100 days after the tumor challenge. The combination treatment group also led to an abscopal effect [134].

## Clinical practice of radiotherapy combined with immunotherapy

Numerous preclinical evidence has established the merit of combining RT with immunotherapy. RT confers immunomodulatory effects and promotes anti-tumor immune responses, thereby augmenting the efficacy of immunotherapy. In light of these findings, many clinical trials evaluating the combined efficacy and safety are presently in progress and have yielded encouraging outcomes.

### Efficacy

Among the clinical trials with published results, RT, in combination with ICB, accounts for the majority. Concurrent chemoradiotherapy is considered the standard treatment for locally advanced esophageal squamous cell carcinoma (ESCC), yielding a median overall survival (OS) time ranging from 18.1 to 19 months. However, for patients who cannot tolerate or decline concurrent chemoradiotherapy, the median OS is 12 months, with RT serving as the primary treatment [148–150]. In one study, 19 patients with locally advanced ESCC (who cannot tolerate or refuse concurrent chemoradiotherapy) received both RT and camrelizumab treatment, with 14 patients (74%) assessed for objective response (CR in 2 patients [11%], partial response [PR] in 12 patients [63%]). The median follow-up time was 31.0 months (95% confidence interval [CI], 27.0–35.1), with median OS and progression-free survival (PFS) times of 16.7 months (95% CI, 5.9–27.9) and 11.7 months (95% CI, 0–30.3), respectively. The 24-month OS and PFS rates were 31.6% and 35.5%, respectively [151]. In a phase I clinical trial, 79 patients with multiple metastatic solid tumors received SBRT followed by PD-1 antibody treatment within 7 days of completing RT. The median follow-up time was 5.5 months. Of the 68 patients evaluated by imaging, the overall objective response rate was 13.2%, with an OS of 9.6 months (95% CI, 6.5 months to undetermined) and a median PFS of 3.1 months (95% CI, 2.9–3.4 months) [152]. Theelen et al. reported the results of the PEMBRO-RT (phase II) and MDACC (phase I/II) trials, which were divided into pembrolizumab alone and RT plus pembrolizumab. In the PEMBRO-RT trial, the first dose of pembrolizumab was given within a week of completing RT, while in the MDACC trial, pembrolizumab was given at the time of the first RT session. A total of 148 patients were included in the pooled analysis, with 76 in the pembrolizumab-alone group and 72 in the RT plus pembrolizumab group. Compared to the alone group, the combination group had a higher best out-of-field (abscopal) response rate (41.7% vs. 19.7%; odds ratio 2.96, 95% CI 1.42–6.20; *p* = 0.0039), best abscopal disease control rate (65.3% vs. 43.4%; 2.51, 1.28–4.91; *p* = 0.0071), longer median PFS (9.0 vs. 4.4 months; hazard ratio 0.67, 95% CI 0.45–0.99; *p* = 0.045), and longer median OS (19.2 vs. 8.7 months; 0.67, 0.54–0.84; *p* = 0.0004) [153]. Apart from the combination with anti-PD-1, a clinical retrospective study of 101 advanced melanoma patients treated with ipilimumab (anti-CTLA-4) showed that the response rate and OS were significantly higher in the 70 patients who received RT during treatment [154]. ICB, such as ipilimumab, is a clinically recommended treatment approach for advanced melanoma [155]. In this study, the addition of RT achieved superior median OS (19 vs. 10 months) and median PFS (5 vs. 3 months) compared to ICB alone [154].

In addition to ICB, there have been few reports of other immunotherapies. A clinical trial enrolled 29 low-grade lymphoma patients to receive in situ vaccination with a TLR9 agonist and local low-dose radiation. Overall, tumor burden decreased in 26 patients, with PR in 7 and CR in 1. Systemic responses outside the irradiated lesions (abscopal effect) occurred in 24 of the 29 patients [156]. In addition, a phase I clinical trial enrolled 12 patients with metastatic renal cell carcinoma and melanoma. The patients received SBRT and high-dose IL-2 with non-irradiated lesion response as the evaluation criterion. One of the 12 patients achieved CR and 7 achieved PR, with an overall response rate of 66.6%, including 1 CR and 4 PR among melanoma patients (response rate of 71.4%) and 3 PR among renal cell carcinoma patients (response rate of 60%) [157]. To minimize adverse reactions, clinical trials of RT combined with modified IL-2 have also been reported for synergistic control of local and distant lesions, such as NHS-IL-2 (NCT00879866) and L19-IL-2 (NCT02086721). Supplementary Table S1 summarizes the clinical trials of RT combined with immunotherapy.

### Safety

While combination therapy has brought promising efficacy, safety remains a concern to be discussed. In a retrospective study exploring the safety and efficacy of ipilimumab combined with stereotactic radiosurgery (SRS) for melanoma brain metastases, only 20% of patients experienced grade 3–4 adverse events (AEs). SRS did not exacerbate typical systemic immune-related AEs associated with ipilimumab, such as enterocolitis, pruritus and hepatitis [158]. All patients receiving RT + camrelizumab reported some form of treatment-related AEs; most were grade 1–2, with no grade 5. The toxicity profile following RT plus camrelizumab was similar to that reported with single-agent therapy. Combination therapy did not increase RT-related toxicity compared to RT alone. The most common AE was immune-related cutaneous capillary hemangioma. Grade 3–4 AEs included lymphopenia, esophagitis, laryngitis and leukopenia [151]. In 18 patients with metastatic urothelial cancer, one patient experienced grade 3 treatment-related lymphopenia after 11 months of treatment. No grade 4–5 treatment-related AEs were observed [159]. In another combination therapy, 6 out of 62 patients experienced grade 3 AEs (pneumonitis in 3 cases, colitis in 2 and hepatic toxicity in 1) [152]. Furthermore, no dose-limiting toxicity related to treatment was observed in the clinical study of RT combined with TLR9 agonist. All patients reported grade 1-2 drug-related AEs, and 8 experienced grade 3 drug-related AEs. No grade 4 or serious drug-related AEs were reported in any patients. The most common treatment-related AE was a flu-like systemic reaction, including malaise, chills, headache, fatigue and fever [156]. It is worth noting that combination therapy may increase immune-related adverse effects at irradiated sites, such as pituitary inflammation when combined with central nervous system radiation, pneumonia when combined with lung radiation, hepatitis when combined with liver irradiation and colitis when combined with intestinal irradiation [160]. The percentages of different grades of AEs are shown in Table 2. In general, radiation therapy combined with immunotherapy is safe and adverse reactions are tolerable. However, larger-scale clinical trials are still needed for further observation.

**Table 2:** Adverse events of radiotherapy combined with immunotherapy.

Types	Cancer species	Treatment protocols	Number of patients (*n*)	Grades (%)			Evaluation criteria	Reference
				1–2	3	4		
AEs	Melanoma brain metastases	SRS + Ipilimumab	46	–	20.0		CTCAE 3.0	[158]
Treatment-related AEs	mCRPC	RT + Ipilimumab	34	–	38.2		CTCAE 3.0	[167]
	Locally advanced ESCC	RT + Camrelizumab	19	100.0	47.4	5.3	CTCAE 4.0	[151]
	Metastatic UC	SBRT + Pembrolizumab	18	83.3	5.5	0	CTCAE 4.0	[159]
	Multiple	SBRT + Pembrolizumab	62	–	9.7	0	CTCAE 4.0	[152]
Treatment-related pulmonary toxicities	NSCLC	RT + Pembrolizumab	97	2.1	0	2.1	CTCAE 4.0	[168]
Drug-related AEs	Low-grade lymphoma	RT + TLR9 agonist	29	100.0	27.6	0	CTCAE 4.0	[156]
Immune-related AEs	Advanced UC	RT + Pembrolizumab	98	–	8.2		CTCAE 5.0	[169]

*CTCAE* the Common Terminology Criteria for Adverse Events, *SRS* stereotactic radiosurgery, *mCRPC* metastatic castration-resistant prostate cancer, *RT* radiotherapy, *SBRT* stereotactic body radiation therapy, *ESCC* esophageal squamous cell carcinoma, *UC* urothelial cancer, *NSCLC* non-small-cell lung cancer.

### Predictive markers of efficacy

Combination therapy with radiation and anti-PD-1/PD-L1 or anti-CTLA-4 has significantly improved efficacy in patients with lung cancer and melanoma. However, a considerable proportion of patients still do not benefit from combination therapy. The clinical features that predict beneficiary populations and the biomarkers of the early anti-tumor immune response are areas that require exploration. The ICD triggered by RT serves as the bridge connecting the body’s immune response, with DAMPs being key molecules in this process. As previously discussed, the escalation of molecules such as HMGB1, HSP70, S100A8/A9, or HMGB1^+^ tumor cells, or EVs carrying HMGB1, HSP70 and HSP90 in the TME, is closely associated with M1 polarization, maturation of DCs, or the recruitment of TANs [65, 77, 92, 107, 108]. These findings suggest that DAMPs may reflect the activation of anti-tumor immune responses and hold the potential to predict the effectiveness of combination therapy. In a clinical trial for advanced NSCLC, SBRT + pembrolizumab only benefited the PD-L1-negative subgroup [161]. Subsequent analysis revealed that responders had higher overall baseline lymphocyte infiltration levels (1.93-fold) [162]. High baseline tumor PD-L1 expression (≥1%), low PD-1^+^CD8^+^ cell density in tumors and a low ratio of PD-L1^+^/CD4^+^ T cells were identified as being associated with better OS [151]. In an analysis of the TCR repertoire in ESCC patients, baseline peripheral CD8^+^ TCR diversity, increased tumor-peripheral Morisita–Horn overlap during treatment and sustained intratumoral T cell clones during treatment were predictive of survival improvement from combination therapy [163]. The predictive value of circulating tumor DNA (ctDNA) has also been reported. Monitoring ctDNA levels during treatment may help predict the treatment response of metastatic urothelial cancer to SBRT + pembrolizumab. In non-responders, ctDNA proportions remained stable or increased, while in responders, ctDNA proportions rapidly decreased [159]. In addition, in assessing the efficacy of radiation in combination with TLR9 agonist, a low baseline percentage of CD4^+^ Tregs and a low initial percentage of proliferating (Ki67^+^) and granzymeB^+^CD8^+^ T cells were associated with better responses [156]. In addition to the biomarkers above, tumor mutation burden, deficient mismatch repair and gut microbiota are also associated with immunotherapy efficacy. However, their predictive value for combination therapy has not been reported. Therefore, it is necessary to consider multiple factors in clinical decision-making to select better patients who benefit from RT combined with immunotherapy.

## Conclusion and perspective

RT combined with immunotherapy has a synergistic effect that enhances the anti-tumor immune response and induces distant effects, providing a new strategy for treating advanced malignancies. Extensive preclinical studies have demonstrated that radiation therapy remodels the TME, reprograms the response status of various immune cells and regulates the immune response by intervening in non-cellular components of the TME. Clinical trials have also shown that combining RT with immunotherapy yields favorable therapeutic outcomes. For tumor patients who do not respond well to immunotherapy, RT may be used as a sensitizing agent to stimulate different steps in the immune response chain.

RT’s reshaping of the TME stands as the cornerstone for the introduction of immunotherapy. Numerous investigations have underscored the role of DAMPs as the mediator linking RT to immunotherapy, with their activation of the cGAS-STING pathway connecting innate and adaptive immunity. However, amidst our enthusiasm for the new opportunities brought forth by combined therapy, attention must be devoted to the challenges that still need to be addressed. (a) The immune suppression and treatment resistance induced by RT cannot be overlooked. Irradiation stimulates the secretion of the cytokine TGF-β, suppressing the activation of DCs and T cells while promoting the expansion of Tregs and immunosuppressive macrophages. Current research has placed relatively less emphasis on mitigating the inhibitory effects of radiation, which holds promise in overcoming treatment resistance and broadening the scope of beneficiaries. (b) The fractionation patterns and total doses of RT for different cancer types remain a challenge at the present stage. Preclinical trials exploring the optimal fractionation patterns of RT in combination with immunotherapy have yielded varying results. The determination of the best fractionation pattern and total dose is currently inconclusive and likely to be associated with tumor types and the accompanying immunotherapeutic agents. In clinical practice, attention must be paid not only to the induction of inflammatory responses but also to the activation of specific anti-tumor immune responses. Regrettably, there is no direct means to assess the impact of different fractionation patterns and doses on the efficacy of immune stimulation. This may necessitate further exploration of more effective biomarkers for assessing efficacy and its impact on the body. (c) RT induces multifaceted responses in the body, and the timing of combining radiation with immunotherapy will be crucial for optimizing efficacy. For example, the release of antigens through radiation is most effective only when Tregs are depleted by CTLA-4 inhibitors [164]. Some studies have suggested that the immediate application of anti-PD-L1 during radiation or post-radiation may be optimal [165, 166]. The best timing should take into account the type of tumor, the mechanisms of action of immunotherapeutic agents and radiation, and their combined impact on the immune microenvironment. Further research is needed to elucidate the timing issues. (d) The immune response generated by radiation is somewhat influenced by the choice of irradiation sites. Organs like the skin, which have a certain degree of external connectivity, exhibit unique immune systems. The liver, due to prolonged exposure to metabolic products, possesses a distinct immune response profile. Moreover, the immune response patterns within the reproductive and central nervous systems diverge considerably. The application of RT to such organs elicits varying degrees of immune response, thereby requiring a comprehensive investigation and analysis to rationally discern the choice of irradiation sites. (e) Identifying the opportune beneficiary groups for RT combined with immunotherapy and seeking prognostic biomarkers is a future exploration field. Combining cell, molecular biology, genomics, and radiomics studies holds the potential to drive forward the clinical application of combination therapy. The development of single-cell multi-omics, artificial intelligence, and deep learning technologies will furnish novel vantage points for us to evaluate RT combined with immunotherapy.

However, RT causes immune suppression, and the effectiveness of combination therapy depends on various factors. Therefore, personalized treatment strategies and thinking should be developed for different tumors in the clinical practice of combined therapy. In the future, further research needs to explore the optimal combined treatment strategies for different tumors, including the selection of irradiated sites, fractionation patterns and total doses, timing windows of combined therapy and biological markers to predict efficacy. With updated evidence from more basic and clinical research, the combination of RT and immunotherapy will become more standardized, safe and precise, bringing more reliable clinical guidance and a brand-new treatment paradigm to cancer patients.

### Supplementary information

Table S1

## References

[1] Ad W, Jm F, Mj L (2020). A guide to cancer immunotherapy: from T cell basic science to clinical practice. Nat Rev Immunol.

[2] Haslam A, Prasad V (2019). Estimation of the percentage of US patients with cancer who are eligible for and respond to checkpoint inhibitor immunotherapy drugs. JAMA Netw Open.

[3] Harding SM, Benci JL, Irianto J, Discher DE, Minn AJ, Greenberg RA (2017). Mitotic progression following DNA damage enables pattern recognition within micronuclei. Nature.

[4] Deng L, Liang H, Xu M, Yang X, Burnette B, Arina A (2014). STING-dependent cytosolic DNA sensing promotes radiation-induced type I interferon-dependent antitumor immunity in immunogenic tumors. Immunity.

[5] Formenti SC, Rudqvist N-P, Golden E, Cooper B, Wennerberg E, Lhuillier C (2018). Radiotherapy induces responses of lung cancer to CTLA-4 blockade. Nat Med.

[6] Speiser DE, Ho P-C, Verdeil G (2016). Regulatory circuits of T cell function in cancer. Nat Rev Immunol.

[7] Mg R, Rl S, Ia W (2006). How TCRs bind MHCs, peptides, and coreceptors. Annu Rev Immunol.

[8] Davis MM, Bjorkman PJ (1988). T-cell antigen receptor genes and T-cell recognition. Nature.

[9] Hou X-L, Wang L, Ding Y-L, Xie Q, Diao H-Y (2016). Current status and recent advances of next generation sequencing techniques in immunological repertoire. Genes Immun.

[10] Jia Q, Wang A, Yuan Y, Zhu B, Long H (2022). Heterogeneity of the tumor immune microenvironment and its clinical relevance. Exp Hematol Oncol.

[11] Reuben A, Gittelman R, Gao J, Zhang J, Yusko EC, Wu C-J (2017). TCR repertoire intratumor heterogeneity in localized lung adenocarcinomas: an association with predicted neoantigen heterogeneity and postsurgical recurrence. Cancer Discov.

[12] Valpione S, Mundra PA, Galvani E, Campana LG, Lorigan P, De Rosa F (2021). The T cell receptor repertoire of tumor infiltrating T cells is predictive and prognostic for cancer survival. Nat Commun.

[13] Chow J, Hoffend NC, Abrams SI, Schwaab T, Singh AK, Muhitch JB (2020). Radiation induces dynamic changes to the T cell repertoire in renal cell carcinoma patients. Proc Natl Acad Sci USA.

[14] Dovedi SJ, Cheadle EJ, Popple AL, Poon E, Morrow M, Stewart R (2017). Fractionated radiation therapy stimulates antitumor immunity mediated by both resident and infiltrating polyclonal T-cell populations when combined with PD-1 blockade. Clin Cancer Res.

[15] Twyman-Saint Victor C, Rech AJ, Maity A, Rengan R, Pauken KE, Stelekati E (2015). Radiation and dual checkpoint blockade activate non-redundant immune mechanisms in cancer. Nature.

[16] Kumar BV, Connors TJ, Farber DL (2018). Human T cell development, localization, and function throughout life. Immunity.

[17] Golstein P, Griffiths GM (2018). An early history of T cell-mediated cytotoxicity. Nat Rev Immunol.

[18] Lai JZ, Zhu YY, Ruan M, Chen L, Zhang QY (2019). Local irradiation sensitized tumors to adoptive T cell therapy via enhancing the cross-priming, homing, and cytotoxicity of antigen-specific CD8 T cells. Front Immunol.

[19] Makarevic A, Rapp C, Dettling S, Reuss D, Jungk C, Abdollahi A (2020). Increased radiation-associated T-cell infiltration in recurrent IDH-mutant glioma. Int J Mol Sci.

[20] Singh AK, Winslow TB, Kermany MH, Goritz V, Heit L, Miller A (2017). A pilot study of stereotactic body radiation therapy combined with cytoreductive nephrectomy for metastatic renal cell carcinoma. Clin Cancer Res.

[21] Herrera FG, Ronet C, Ochoa de Olza M, Barras D, Crespo I, Andreatta M (2022). Low-dose radiotherapy reverses tumor immune desertification and resistance to immunotherapy. Cancer Discov.

[22] Chen J, Cao Y, Markelc B, Kaeppler J, Vermeer JA, Muschel RJ (2019). Type I IFN protects cancer cells from CD8+ T cell-mediated cytotoxicity after radiation. J Clin Invest.

[23] Jarosz-Biej M, Smolarczyk R, Cichoń T, Drzyzga A, Czapla J, Urbaś Z (2020). Brachytherapy in a single dose of 10Gy as an ‘in situ’ vaccination. Int J Mol Sci.

[24] Bendelac A, Rivera MN, Park SH, Roark JH (1997). Mouse CD1-specific NK1 T cells: development, specificity, and function. Annu Rev Immunol.

[25] Wu D, Xing G-W, Poles MA, Horowitz A, Kinjo Y, Sullivan B (2005). Bacterial glycolipids and analogs as antigens for CD1d-restricted NKT cells. Proc Natl Acad Sci USA.

[26] Kobayashi K, Tanaka Y, Horiguchi S, Yamamoto S, Toshinori N, Sugimoto A (2010). The effect of radiotherapy on NKT cells in patients with advanced head and neck cancer. Cancer Immunol Immunother.

[27] Mueller SN, Gebhardt T, Carbone FR, Heath WR (2013). Memory T cell subsets, migration patterns, and tissue residence. Annu Rev Immunol.

[28] Gide TN, Quek C, Menzies AM, Tasker AT, Shang P, Holst J (2019). Distinct immune cell populations define response to Anti-PD-1 monotherapy and anti-PD-1/anti-CTLA-4 combined therapy. Cancer Cell.

[29] Huang Q, Wu X, Wang Z, Chen X, Wang L, Lu Y (2022). The primordial differentiation of tumor-specific memory CD8+ T cells as bona fide responders to PD-1/PD-L1 blockade in draining lymph nodes. Cell.

[30] Chen JL, Pan CK, Huang YS, Tsai CY, Wang CW, Lin YL (2021). Evaluation of antitumor immunity by a combination treatment of high-dose irradiation, anti-PDL1, and anti-angiogenic therapy in murine lung tumors. Cancer Immunol Immunother.

[31] McGee HM, Daly ME, Azghadi S, Stewart SL, Oesterich L, Schlom J (2018). Stereotactic ablative radiation therapy induces systemic differences in peripheral blood immunophenotype dependent on irradiated site. Int J Radiat Oncol Biol Phys.

[32] Wherry EJ, Kurachi M (2015). Molecular and cellular insights into T cell exhaustion. Nat Rev Immunol.

[33] Beltra J-C, Manne S, Abdel-Hakeem MS, Kurachi M, Giles JR, Chen Z (2020). Developmental relationships of four exhausted CD8+ T cell subsets reveals underlying transcriptional and epigenetic landscape control mechanisms. Immunity.

[34] Wang B, Hu J, Zhang J, Zhao L (2022). Radiation therapy regulates TCF-1 to maintain CD8+T cell stemness and promotes anti-tumor immunotherapy. Int Immunopharmacol.

[35] Li W, Lu L, Lu J, Wang X, Yang C, Jin J (2020). cGAS-STING-mediated DNA sensing maintains CD8+ T cell stemness and promotes antitumor T cell therapy. Sci Transl Med.

[36] Curotto de Lafaille MA, Lafaille JJ (2009). Natural and adaptive foxp3+ regulatory T cells: more of the same or a division of labor?. Immunity.

[37] Yoshida K, Okamoto M, Sasaki J, Kuroda C, Ishida H, Ueda K (2020). Anti-PD-1 antibody decreases tumour-infiltrating regulatory T cells. BMC Cancer.

[38] Kamada T, Togashi Y, Tay C, Ha D, Sasaki A, Nakamura Y (2019). PD-1+ regulatory T cells amplified by PD-1 blockade promote hyperprogression of cancer. Proc Natl Acad Sci USA.

[39] Dutt S, Atallah MB, Minamida Y, Filatenkov A, Jensen KP, Iliopoulou BP (2018). Accelerated, but not conventional, radiotherapy of murine B-cell lymphoma induces potent T cell-mediated remissions. Blood Adv.

[40] Beauford SS, Kumari A, Garnett-Benson C (2020). Ionizing radiation modulates the phenotype and function of human CD4+ induced regulatory T cells. BMC Immunol.

[41] Schaue D, Ratikan JA, Iwamoto KS, McBride WH (2012). Maximizing tumor immunity with fractionated radiation. Int J Radiat Oncol Biol Phys.

[42] Boss MK, Watts R, Harrison LG, Hopkins S, Chow L, Trageser E (2022). Immunologic effects of stereotactic body radiotherapy in dogs with spontaneous tumors and the impact of intratumoral OX40/TLR agonist immunotherapy. Int J Mol Sci.

[43] Wang M, Gou X, Wang L (2012). Protein kinase B promotes radiation-induced regulatory T cell survival in bladder carcinoma. Scand J Immunol.

[44] Yoo JK, Cho JH, Lee SW, Sung YC (2002). IL-12 provides proliferation and survival signals to murine CD4+ T cells through phosphatidylinositol 3-kinase/Akt signaling pathway. J Immunol Balt Md.

[45] De Martino M, Daviaud C, Diamond JM, Kraynak J, Alard A, Formenti SC (2021). Activin A promotes regulatory T-cell–mediated immunosuppression in irradiated breast cancer. Cancer Immunol Res.

[46] Morianos I, Papadopoulou G, Semitekolou M, Xanthou G (2019). Activin-A in the regulation of immunity in health and disease. J Autoimmun.

[47] Ono M (2020). Control of regulatory T-cell differentiation and function by T-cell receptor signalling and Foxp3 transcription factor complexes. Immunology.

[48] Leone RD, Powell JD (2020). Metabolism of immune cells in cancer. Nat Rev Cancer.

[49] Chang C-H, Curtis JD, Maggi LB, Faubert B, Villarino AV, O’Sullivan D (2013). Posttranscriptional control of T cell effector function by aerobic glycolysis. Cell.

[50] Jacobs SR, Herman CE, Maciver NJ, Wofford JA, Wieman HL, Hammen JJ (2008). Glucose uptake is limiting in T cell activation and requires CD28-mediated Akt-dependent and independent pathways. J Immunol Balt Md.

[51] Madden MZ, Rathmell JC (2021). The complex integration of T-cell metabolism and immunotherapy. Cancer Discov.

[52] Li H-H, Wang Y-W, Chen R, Zhou B, Ashwell JD, Fornace AJ (2015). Ionizing radiation impairs T cell activation by affecting metabolic reprogramming. Int J Biol Sci.

[53] Verbist KC, Guy CS, Milasta S, Liedmann S, Kamiński MM, Wang R (2016). Metabolic maintenance of cell asymmetry following division in activated T lymphocytes. Nature.

[54] Wang J-S, Wang H-J, Qian H-L (2018). Biological effects of radiation on cancer cells. Mil Med Res.

[55] Sena LA, Li S, Jairaman A, Prakriya M, Ezponda T, Hildeman DA (2013). Mitochondria are required for antigen-specific T cell activation through reactive oxygen species signaling. Immunity.

[56] Maj T, Wang W, Crespo J, Zhang H, Wang W, Wei S (2017). Oxidative stress controls regulatory T cell apoptosis and suppressor activity and PD-L1-blockade resistance in tumor. Nat Immunol.

[57] Yu X, Lao Y, Teng X-L, Li S, Zhou Y, Wang F (2018). SENP3 maintains the stability and function of regulatory T cells via BACH2 deSUMOylation. Nat Commun.

[58] Angelin A, Gil-de-Gómez L, Dahiya S, Jiao J, Guo L, Levine MH (2017). Foxp3 reprograms T cell metabolism to function in low-glucose, high-lactate environments. Cell Metab.

[59] Mantovani A, Allavena P, Marchesi F, Garlanda C (2022). Macrophages as tools and targets in cancer therapy. Nat Rev Drug Discov.

[60] Sica A, Mantovani A (2012). Macrophage plasticity and polarization: in vivo veritas. J Clin Invest.

[61] Mantovani A, Sica A, Sozzani S, Allavena A, Vecchi A, Locati M (2004). The chemokine system in diverse forms of macrophage activation and polarization. Trends Immunol.

[62] Gao J, Liang Y, Wang L (2022). Shaping polarization of tumor-associated macrophages in cancer immunotherapy. Front Immunol.

[63] Klug F, Prakash H, Huber PE, Seibel T, Bender N, Halama N (2013). Low-dose irradiation programs macrophage differentiation to an iNOS^+^/M1 phenotype that orchestrates effective T cell immunotherapy. Cancer Cell.

[64] Zhang J, Liu X, Wan C, Liu Y, Wang Y, Meng C (2020). NLRP3 inflammasome mediates M1 macrophage polarization and IL-1β production in inflammatory root resorption. J Clin Periodontol.

[65] Zhu L, Hu S, Chen Q, Zhang H, Fu J, Zhou Y (2021). Macrophage contributes to radiation-induced anti-tumor abscopal effect on transplanted breast cancer by HMGB1/TNF-α signaling factors. Int J Biol Sci.

[66] Anuranjani, Bala M (2014). Concerted action of Nrf2-ARE pathway, MRN complex, HMGB1 and inflammatory cytokines—implication in modification of radiation damage. Redox Biol.

[67] Karuppagounder V, Giridharan VV, Arumugam S, Sreedhar R, Palaniyandi SS, Krishnamurthy P (2016). Modulation of macrophage polarization and HMGB1-TLR2/TLR4 cascade plays a crucial role for cardiac remodeling in senescence-accelerated prone mice. PLoS One.

[68] Guo C, Guo D, Fang L, Sang T, Wu J, Guo C (2021). Ganoderma lucidum polysaccharide modulates gut microbiota and immune cell function to inhibit inflammation and tumorigenesis in colon. Carbohydr Polym.

[69] Liu S, Lu C, Liu Y, Zhou X, Sun L, Gu Q (2018). Hyperbaric oxygen alleviates the inflammatory response induced by LPS through inhibition of NF-κB/MAPKs-CCL2/CXCL1 signaling pathway in cultured astrocytes. Inflammation.

[70] Moreira D, Sampath S, Won H, White SV, Su Y-L, Alcantara M (2021). Myeloid cell–targeted STAT3 inhibition sensitizes head and neck cancers to radiotherapy and T cell–mediated immunity. J Clin Invest.

[71] Jin Y, Kang Y, Wang M, Wu B, Su B, Yin H (2022). Targeting polarized phenotype of microglia via IL6/JAK2/STAT3 signaling to reduce NSCLC brain metastasis. Signal Transduct Target Ther.

[72] Palucka K, Banchereau J (2012). Cancer immunotherapy via dendritic cells. Nat Rev Cancer.

[73] Wculek SK, Cueto FJ, Mujal AM, Melero I, Krummel MF, Sancho D (2020). Dendritic cells in cancer immunology and immunotherapy. Nat Rev Immunol.

[74] Frey B, Rückert M, Weber J, Mayr X, Derer A, Lotter M (2017). Hypofractionated irradiation has immune stimulatory potential and induces a timely restricted infiltration of immune cells in colon cancer tumors. Front Immunol.

[75] Philippou Y, Sjoberg HT, Murphy E, Alyacoubi S, Jones KI, Gordon-Weeks AN (2020). Impacts of combining anti-PD-L1 immunotherapy and radiotherapy on the tumour immune microenvironment in a murine prostate cancer model. Br J Cancer.

[76] Keam SP, Halse H, Nguyen T, Wang M, Van Kooten Losio N, Mitchell C (2020). High dose-rate brachytherapy of localized prostate cancer converts tumors from cold to hot. J Immunother Cancer.

[77] Chang MC, Chen YL, Lin HW, Chiang YC, Chang CF, Hsieh SF (2018). Irradiation enhances abscopal anti-tumor effects of antigen-specific immunotherapy through regulating tumor microenvironment. Mol Ther.

[78] Zheng J, Mo J, Zhu T, Zhuo W, Yi Y, Hu S (2020). Comprehensive elaboration of the cGAS-STING signaling axis in cancer development and immunotherapy. Mol Cancer.

[79] Hong S, Bi M, Yu H, Yan Z, Wang H (2020). Radiation therapy enhanced therapeutic efficacy of anti-PD1 against gastric cancer. J Radiat Res.

[80] Veglia F, Sanseviero E, Gabrilovich DI (2021). Myeloid-derived suppressor cells in the era of increasing myeloid cell diversity. Nat Rev Immunol.

[81] Tcyganov E, Mastio J, Chen E, Gabrilovich DI (2018). Plasticity of myeloid-derived suppressor cells in cancer. Curr Opin Immunol.

[82] Corzo CA, Condamine T, Lu L, Cotter MJ, Youn J-I, Cheng P (2010). HIF-1α regulates function and differentiation of myeloid-derived suppressor cells in the tumor microenvironment. J Exp Med.

[83] Kumar V, Patel S, Tcyganov E, Gabrilovich DI (2016). The nature of myeloid-derived suppressor cells in the tumor microenvironment. Trends Immunol.

[84] Zhang S, Ma X, Zhu C, Liu L, Wang G, Yuan X (2016). The role of myeloid-derived suppressor cells in patients with solid tumors: a meta-analysis. PLoS One.

[85] Riva M, Wouters R, Nittner D, Ceuster J, Sterpin E, Giovannoni R, et al. Radiation dose-escalation and dose-fractionation modulate the immune microenvironment, cancer stem cells and vasculature in experimental high-grade gliomas. J Neurosurg Sci. 2023;67:55–65. 10.23736/S0390-5616.20.05060-2 33056947

[86] Deng L, Liang H, Burnette B, Beckett M, Darga T, Weichselbaum RR (2014). Irradiation and anti-PD-L1 treatment synergistically promote antitumor immunity in mice. J Clin Invest.

[87] Gabrilovich DI, Nagaraj S (2009). Myeloid-derived suppressor cells as regulators of the immune system. Nat Rev Immunol.

[88] Wu CJ, Tsai YT, Lee IJ, Wu PY, Lu LS, Tsao WS (2018). Combination of radiation and interleukin 12 eradicates large orthotopic hepatocellular carcinoma through immunomodulation of tumor microenvironment. Oncoimmunology.

[89] Ohl K, Tenbrock K (2018). Reactive oxygen species as regulators of MDSC-mediated immune suppression. Front Immunol.

[90] Németh T, Sperandio M, Mócsai A (2020). Neutrophils as emerging therapeutic targets. Nat Rev Drug Discov.

[91] Hedrick CC, Malanchi I (2022). Neutrophils in cancer: heterogeneous and multifaceted. Nat Rev Immunol.

[92] Krombach J, Hennel R, Brix N, Orth M, Schoetz U, Ernst A (2019). Priming anti-tumor immunity by radiotherapy: Dying tumor cell-derived DAMPs trigger endothelial cell activation and recruitment of myeloid cells. Oncoimmunology.

[93] Liu Q, Hao Y, Du R, Hu D, Xie J, Zhang J (2021). Radiotherapy programs neutrophils to an antitumor phenotype by inducing mesenchymal-epithelial transition. Transl Lung Cancer Res.

[94] Ali M, Fulci G, Grigalavicius M, Pulli B, Li A, Wojtkiewicz GR (2022). Myeloperoxidase exerts anti-tumor activity in glioma after radiotherapy. Neoplasia.

[95] Grisaru-Tal S, Itan M, Klion AD, Munitz A (2020). A new dawn for eosinophils in the tumour microenvironment. Nat Rev Cancer.

[96] Cheng JN, Luo W, Sun C, Jin Z, Zeng X, Alexander PB (2021). Radiation-induced eosinophils improve cytotoxic T lymphocyte recruitment and response to immunotherapy. Sci Adv.

[97] Biswas SK (2015). Metabolic reprogramming of immune cells in cancer progression. Immunity.

[98] Certo M, Tsai C-H, Pucino V, Ho P-C, Mauro C (2021). Lactate modulation of immune responses in inflammatory versus tumour microenvironments. Nat Rev Immunol.

[99] Yang X, Lu Y, Hang J, Zhang J, Zhang T, Huo Y (2020). Lactate-modulated immunosuppression of myeloid-derived suppressor cells contributes to the radioresistance of pancreatic cancer. Cancer Immunol Res.

[100] Lai Y-C, Hsieh C-Y, Lu K-Y, Sung C-H, Ho H-Y, Cheng M-L (2021). Monitoring early glycolytic flux alterations following radiotherapy in cancer and immune cells: hyperpolarized carbon-13 magnetic resonance imaging study. Metabolites.

[101] Hambardzumyan D, Gutmann DH, Kettenmann H (2016). The role of microglia and macrophages in glioma maintenance and progression. Nat Neurosci.

[102] Brand A, Singer K, Koehl GE, Kolitzus M, Schoenhammer G, Thiel A (2016). LDHA-associated lactic acid production blunts tumor immunosurveillance by T and NK cells. Cell Metab.

[103] Kumagai S, Koyama S, Itahashi K, Tanegashima T, Lin Y, Togashi Y (2022). Lactic acid promotes PD-1 expression in regulatory T cells in highly glycolytic tumor microenvironments. Cancer Cell.

[104] Théry C, Ostrowski M, Segura E (2009). Membrane vesicles as conveyors of immune responses. Nat Rev Immunol.

[105] Xie F, Zhou X, Fang M, Li H, Su P, Tu Y, et al. Extracellular vesicles in cancer immune microenvironment and cancer immunotherapy. Adv Sci. 2019;6:1901779. 10.1002/advs.201901779 PMC6918121 31871860

[106] Marar C, Starich B, Wirtz D (2021). Extracellular vesicles in immunomodulation and tumor progression. Nat Immunol.

[107] Jella KK, Nasti TH, Li Z, Lawson DH, Switchenko JM, Ahmed R (2020). Exosome-containing preparations from postirradiated mouse melanoma cells delay melanoma growth in vivo by a natural killer cell–dependent mechanism. Int J Radiat Oncol.

[108] Lin W, Xu Y, Chen X, Liu J, Weng Y, Zhuang Q (2020). Radiation-induced small extracellular vesicles as “carriages” promote tumor antigen release and trigger antitumor immunity. Theranostics.

[109] Stary V, Wolf B, Unterleuthner D, List J, Talic M, Laengle J (2020). Short-course radiotherapy promotes pro-inflammatory macrophages via extracellular vesicles in human rectal cancer. J Immunother Cancer.

[110] Diamond JM, Vanpouille-Box C, Spada S, Rudqvist N-P, Chapman JR, Ueberheide BM (2018). Exosomes shuttle TREX1-sensitive IFN-stimulatory dsDNA from irradiated cancer cells to DCs. Cancer Immunol Res.

[111] Han C, Godfrey V, Liu Z, Han Y, Liu L, Peng H (2021). The AIM2 and NLRP3 inflammasomes trigger IL-1–mediated antitumor effects during radiation. Sci Immunol.

[112] Chen F, Chen J, Yang L, Liu J, Zhang X, Zhang Y (2019). Extracellular vesicle-packaged HIF-1α-stabilizing lncRNA from tumour-associated macrophages regulates aerobic glycolysis of breast cancer cells. Nat Cell Biol.

[113] Wu C-H, Li J, Li L, Sun J, Fabbri M, Wayne AS (2019). Extracellular vesicles derived from natural killer cells use multiple cytotoxic proteins and killing mechanisms to target cancer cells. J Extracell Vesicles.

[114] Pardoll DM (2012). The blockade of immune checkpoints in cancer immunotherapy. Nat Rev Cancer.

[115] Cogdill AP, Andrews MC, Wargo JA (2017). Hallmarks of response to immune checkpoint blockade. Br J Cancer.

[116] Yang C-Y, Lin M-W, Chang Y-L, Wu C-T, Yang P-C (2016). Programmed cell death-ligand 1 expression is associated with a favourable immune microenvironment and better overall survival in stage I pulmonary squamous cell carcinoma. Eur J Cancer.

[117] Mills BN, Qiu H, Drage MG, Chen C, Mathew JS, Garrett-Larsen J (2022). Modulation of the human pancreatic ductal adenocarcinoma immune microenvironment by stereotactic body radiotherapy. Clin Cancer Res.

[118] Kikuchi M, Clump DA, Srivastava RM, Sun L, Zeng D, Diaz-Perez JA (2017). Preclinical immunoPET/CT imaging using Zr-89-labeled anti-PD-L1 monoclonal antibody for assessing radiation-induced PD-L1 upregulation in head and neck cancer and melanoma. Oncoimmunology.

[119] Azad A, Yin Lim S, D’Costa Z, Jones K, Diana A, Sansom OJ (2017). PD-L1 blockade enhances response of pancreatic ductal adenocarcinoma to radiotherapy. EMBO Mol Med.

[120] Grapin M, Richard C, Limagne E, Boidot R, Morgand V, Bertaut A (2019). Optimized fractionated radiotherapy with anti-PD-L1 and anti-TIGIT: a promising new combination. J Immunother Cancer.

[121] Kumazawa T, Mori Y, Sato H, Permata TBM, Uchihara Y, Noda SE (2022). Expression of non-homologous end joining factor, Ku80, is negatively correlated with PD-L1 expression in cancer cells after X-ray irradiation. Oncol Lett.

[122] Oweida A, Hararah MK, Phan A, Binder D, Bhatia S, Lennon S (2018). Resistance to radiotherapy and PD-L1 blockade is mediated by TIM-3 upregulation and regulatory T-cell infiltration. Clin Cancer Res.

[123] Manieri NA, Chiang EY, Grogan JL (2017). TIGIT: a key inhibitor of the cancer immunity cycle. Trends Immunol.

[124] Zhu C, Anderson AC, Schubart A, Xiong H, Imitola J, Khoury SJ (2005). The Tim-3 ligand galectin-9 negatively regulates T helper type 1 immunity. Nat Immunol.

[125] Maruhashi T, Sugiura D, Okazaki I-M, Shimizu K, Maeda TK, Ikubo J (2022). Binding of LAG-3 to stable peptide-MHC class II limits T cell function and suppresses autoimmunity and anti-cancer immunity. Immunity.

[126] Matsuzaki J, Gnjatic S, Mhawech-Fauceglia P, Beck A, Miller A, Tsuji T (2010). Tumor-infiltrating NY-ESO-1-specific CD8+ T cells are negatively regulated by LAG-3 and PD-1 in human ovarian cancer. Proc Natl Acad Sci USA.

[127] Donlon NE, Davern M, O’Connell F, Sheppard A, Heeran A, Bhardwaj A (2022). Impact of radiotherapy on the immune landscape in oesophageal adenocarcinoma. World J Gastroenterol.

[128] Brand DH, Kirby AM, Yarnold JR, Somaiah N (2022). How low can you go? The radiobiology of hypofractionation. Clin Oncol.

[129] Thariat J, Hannoun-Levi J-M, Sun Myint A, Vuong T, Gérard J-P (2013). Past, present, and future of radiotherapy for the benefit of patients. Nat Rev Clin Oncol.

[130] Grimm J, Marks LB, Jackson A, Kavanagh BD, Xue J, Yorke E (2021). High dose per fraction, Hypofractionated Treatment Effects in the Clinic (HyTEC): an overview. Int J Radiat Oncol Biol Phys.

[131] Gao H, Dong Z, Gong X, Dong J, Zhang Y, Wei W (2018). Effects of various radiation doses on induced T-helper cell differentiation and related cytokine secretion. J Radiat Res.

[132] Barsoumian HB, Ramapriyan R, Younes AI, Caetano MS, Menon H, Comeaux NI (2020). Low-dose radiation treatment enhances systemic antitumor immune responses by overcoming the inhibitory stroma. J Immunother Cancer.

[133] Mills BN, Connolly KA, Ye J, Murphy JD, Uccello TP, Han BJ (2019). Stereotactic body radiation and interleukin-12 combination therapy eradicates pancreatic tumors by repolarizing the immune microenvironment. Cell Rep.

[134] Chen WY, Chen YL, Lin HW, Chang CF, Huang BS, Sun WZ (2021). Stereotactic body radiation combined with oncolytic vaccinia virus induces potent anti-tumor effect by triggering tumor cell necroptosis and DAMPs. Cancer Lett.

[135] Salmon H, Remark R, Gnjatic S, Merad M (2019). Host tissue determinants of tumour immunity. Nat Rev Cancer.

[136] Riva M, Wouters R, Sterpin E, Giovannoni R, Boon L, Himmelreich U (2021). Radiotherapy, temozolomide, and antiprogrammed cell death protein 1 treatments modulate the immune microenvironment in experimental high-grade glioma. Neurosurgery.

[137] Wang J, Dai Z, Miao Y, Zhao T, Gan J, Zhao C (2021). Carbon ion (12C6+) irradiation induces the expression of Klrk1 in lung cancer and optimizes the tumor microenvironment based on the NKG2D/NKG2D-Ls pathway. Cancer Lett.

[138] Yu J, Green MD, Li S, Sun Y, Journey SN, Choi JE (2021). Liver metastasis restrains immunotherapy efficacy via macrophage-mediated T cell elimination. Nat Med.

[139] Prakash H, Klug F, Nadella V, Mazumdar V, Schmitz-Winnenthal H, Umansky L (2016). Low doses of gamma irradiation potentially modifies immunosuppressive tumor microenvironment by retuning tumor-associated macrophages: lesson from insulinoma. Carcinogenesis.

[140] Pereira PMR, Edwards KJ, Mandleywala K, Carter LM, Escorcia FE, Campesato LF (2020). iNOS regulates the therapeutic response of pancreatic cancer cells to radiotherapy. Cancer Res.

[141] Wilkins A, Fontana E, Nyamundanda G, Ragulan C, Patil Y, Mansfield D (2021). Differential and longitudinal immune gene patterns associated with reprogrammed microenvironment and viral mimicry in response to neoadjuvant radiotherapy in rectal cancer. J Immunother Cancer.

[142] Mole RH (1953). Whole body irradiation; radiobiology or medicine?. Br J Radio.

[143] Dewan MZ, Galloway AE, Kawashima N, Dewyngaert JK, Babb JS, Formenti SC (2009). Fractionated but not single-dose radiotherapy induces an immune-mediated abscopal effect when combined with anti-CTLA-4 antibody. Clin Cancer Res.

[144] Murty S, Haile ST, Beinat C, Aalipour A, Alam IS, Murty T (2020). Intravital imaging reveals synergistic effect of CAR T-cells and radiation therapy in a preclinical immunocompetent glioblastoma model. Oncoimmunology.

[145] Luo M, Liu Z, Zhang X, Han C, Samandi LZ, Dong C (2019). Synergistic STING activation by PC7A nanovaccine and ionizing radiation improves cancer immunotherapy. J Control Release.

[146] Pilones KA, Charpentier M, Garcia-Martinez E, Daviaud C, Kraynak J, Aryankalayil J (2020). Radiotherapy cooperates with IL15 to induce antitumor immune responses. Cancer Immunol Res.

[147] Lichty BD, Breitbach CJ, Stojdl DF, Bell JC (2014). Going viral with cancer immunotherapy. Nat Rev Cancer.

[148] Cooper JS, Guo MD, Herskovic A, Macdonald JS, Martenson JA, Al-Sarraf M (1999). Chemoradiotherapy of locally advanced esophageal cancer: long-term follow-up of a prospective randomized trial (RTOG 85-01). Radiation Therapy Oncology Group. JAMA.

[149] Minsky BD, Pajak TF, Ginsberg RJ, Pisansky TM, Martenson J, Komaki R (2002). INT 0123 (Radiation Therapy Oncology Group 94-05) phase III trial of combined-modality therapy for esophageal cancer: high-dose versus standard-dose radiation therapy. J Clin Oncol.

[150] Suntharalingam M, Winter K, Ilson D, Dicker AP, Kachnic L, Konski A (2017). Effect of the addition of cetuximab to paclitaxel, cisplatin, and radiation therapy for patients with esophageal cancer: The NRG Oncology RTOG 0436 Phase 3 Randomized Clinical Trial. JAMA Oncol.

[151] Zhang W, Yan C, Gao X, Li X, Cao F, Zhao G (2021). Safety and feasibility of radiotherapy plus camrelizumab for locally advanced esophageal squamous cell carcinoma. Oncologist.

[152] Luke JJ, Lemons JM, Karrison TG, Pitroda SP, Melotek JM, Zha Y (2018). Safety and clinical activity of pembrolizumab and multisite stereotactic body radiotherapy in patients with advanced solid tumors. J Clin Oncol.

[153] Theelen WSME, Chen D, Verma V, Hobbs BP, Peulen HMU, Aerts JGJV (2021). Pembrolizumab with or without radiotherapy for metastatic non-small-cell lung cancer: a pooled analysis of two randomised trials. Lancet Respir Med.

[154] Koller KM, Mackley HB, Liu J, Wagner H, Talamo G, Schell TD (2017). Improved survival and complete response rates in patients with advanced melanoma treated with concurrent ipilimumab and radiotherapy versus ipilimumab alone. Cancer Biol Ther.

[155] Curti BD, Faries MB (2021). Recent advances in the treatment of melanoma. N Engl J Med.

[156] Frank MJ, Reagan PM, Bartlett NL, Gordon LI, Friedberg JW, Czerwinski DK (2018). In situ vaccination with a TLR9 agonist and local low-dose radiation induces systemic responses in untreated indolent lymphoma. Cancer Discov.

[157] Seung SK, Curti BD, Crittenden M, Walker E, Coffey T, Siebert JC (2012). Phase 1 study of stereotactic body radiotherapy and interleukin-2-tumor and immunological responses. Sci Transl Med.

[158] Kiess AP, Wolchok JD, Barker CA, Postow MA, Tabar V, Huse JT (2015). Stereotactic radiosurgery for melanoma brain metastases in patients receiving ipilimumab: safety profile and efficacy of combined treatment. Int J Radiat Oncol Biol Phys.

[159] Sundahl N, Vandekerkhove G, Decaestecker K, Meireson A, De Visschere P, Fonteyne V (2019). Randomized phase 1 trial of pembrolizumab with sequential versus concomitant stereotactic body radiotherapy in metastatic urothelial carcinoma. Eur Urol.

[160] Sharabi AB, Lim M, DeWeese TL, Drake CG (2015). Radiation and checkpoint blockade immunotherapy: radiosensitisation and potential mechanisms of synergy. Lancet Oncol.

[161] Theelen W, Peulen HMU, Lalezari F, van der Noort V, de Vries JF, Aerts J (2019). Effect of pembrolizumab after stereotactic body radiotherapy vs pembrolizumab alone on tumor response in patients with advanced non-small cell lung cancer: results of the PEMBRO-RT Phase 2 Randomized Clinical Trial. JAMA Oncol.

[162] van der Woude LL, Gorris MAJ, Wortel IMN, Creemers JHA, Verrijp K, Monkhorst K (2022). Tumor microenvironment shows an immunological abscopal effect in patients with NSCLC treated with pembrolizumab-radiotherapy combination. J Immunother Cancer.

[163] Yan C, Ma X, Guo Z, Wei X, Han D, Zhang T (2022). Time-spatial analysis of T cell receptor repertoire in esophageal squamous cell carcinoma patients treated with combined radiotherapy and PD-1 blockade. Oncoimmunology.

[164] Young KH, Baird JR, Savage T, Cottam B, Friedman D, Bambina S (2016). Optimizing timing of immunotherapy improves control of tumors by hypofractionated radiation therapy. PLoS One.

[165] Dovedi SJ, Illidge TM (2015). The antitumor immune response generated by fractionated radiation therapy may be limited by tumor cell adaptive resistance and can be circumvented by PD-L1 blockade. Oncoimmunology.

[166] Dovedi SJ, Adlard AL, Lipowska-Bhalla G, McKenna C, Jones S, Cheadle EJ (2014). Acquired resistance to fractionated radiotherapy can be overcome by concurrent PD-L1 blockade. Cancer Res.

[167] Slovin SF, Higano CS, Hamid O, Tejwani S, Harzstark A, Alumkal JJ (2013). Ipilimumab alone or in combination with radiotherapy in metastatic castration-resistant prostate cancer: results from an open-label, multicenter phase I/II study. Ann Oncol.

[168] Shaverdian N, Lisberg AE, Bornazyan K, Veruttipong D, Goldman JW, Formenti SC (2017). Previous radiotherapy and the clinical activity and toxicity of pembrolizumab in the treatment of non-small-cell lung cancer: a secondary analysis of the KEYNOTE-001 phase 1 trial. Lancet Oncol.

[169] Fukushima H, Kijima T, Fukuda S, Moriyama S, Uehara S, Yasuda Y (2020). Impact of radiotherapy to the primary tumor on the efficacy of pembrolizumab for patients with advanced urothelial cancer: a preliminary study. Cancer Med.

