# Supplementary material for: Radiotherapy remodels the tumor microenvironment for enhancing immunotherapeutic sensitivity
Source: Cell Death Dis. 2023 Oct 13;14(10):679. doi: 10.1038/s41419-023-06211-2 (PMC10575861; doi:10.1038/s41419-023-06211-2)
Supplement: Supplementary file 1 — Table S1 [file 41419_2023_6211_MOESM1_ESM.docx]

**Table S1 Clinical trials combining radiotherapy with immunotherapy**

| **Immunotherapy** | **Radiotherapy** | **Cancer species** | **Number of patients (n)** | **Phases** | **Status** | **NCT ID** |
| --- | --- | --- | --- | --- | --- | --- |
| Anti-PD-1 | HDCRT | Advanced malignancies | 21 | I | Completed | NCT02987166 |
|  | SBRT | Stage IV NSCLC | 29 | I | Completed | NCT03812549 |
|  | SBRT | Metastatic UC | 18 | I | Completed | NCT02826564 |
|  | RT | Advanced HNC | 15 | II | Recruiting | NCT04454489 |
|  | Short-course RT | Early low rectal cancer | 34 | II | Recruiting | NCT05555888 |
|  | HDRT | Prostate cancer | 44 | I/II | Recruiting | NCT03543189 |
|  | Diffusing alpha-emitters RT (DaRT) | HNSCC | 48 | NA | Recruiting | NCT05047094 |
|  | RT | HNSCC | 114 | II | Recruiting | NCT03383094 |
|  | SRT | Glioblastoma | 10 | I/II | Recruiting | NCT04977375 |
|  | RT | Oropharyngeal cancer | 590 | II/III | Recruiting | NCT03952585 |
|  | RT | Melanoma | 168 | II | Recruiting | NCT04594187 |
|  | SBRT | NPC | 188 | III | Recruiting | NCT04944914 |
|  | RT | STS | 12 | Early Phase I | Recruiting | NCT05488366 |
|  | SBRT, IMRT | NPC | 38 | II | Recruiting | NCT05431764 |
|  | RT | ESCC | 26 | I/II | Recruiting | NCT05176002 |
|  | LDRT | Advanced malignant tumors | 20 | NA | Recruiting | NCT05547282 |
|  | SBRT | HNSCC | 102 | II | Recruiting | NCT03546582 |
|  | RT | Melanoma | 20 | Early Phase I | Recruiting | NCT0458I382 |
|  | SBRT | Stage IV prostate cancer | 90 | II | Recruiting | NCT05655715 |
|  | SBRT, LDRT | NSCLC | 124 | II | Recruiting | NCT05798845 |
|  | SBRT | HNSCC | 28 | II | Recruiting | NCT04938609 |
|  | RT | EC | 56 | NA | Recruiting | NCT06009705 |
|  | RT | HNSCC | 20 | I/II | Recruiting | NCT03317327 |
|  | RT | NSCLC | 30 | NA | Recruiting | NCT03705806 |
|  | RT | HNSCC | 316 | II | Recruiting | NCT04523883 |
|  | SBRT | Epidural spinal tumors | 10 | Early Phase I | Recruiting | NCT05204290 |
|  | SBRT | Bladder cancer | 33 | II | Recruiting | NCT05241340 |
|  | EBRT | HNSCC | 45 | I | Recruiting | NCT04754321 |
|  | RT | Stage IV NSCLC | 40 | II | Recruiting | NCT05691829 |
|  | CIRT | NSCLS, HNSCC, melanoma, UC | 27 | II | Recruiting | NCT05229614 |
|  | Short-course RT | Locally advanced rectal cancer | 130 | II | Recruiting | NCT04518280 |
|  | RT | Cutaneous squamous cell carcinoma | 34 | II | Recruiting | NCT05574101 |
|  | RT | Advanced solid tumors | 55 | II | Recruiting | NCT05097781 |
|  | SRS | Meningioma | 37 | II | Recruiting | NCT04659811 |
|  | SBRT | ESCC | 40 | II | Recruiting | NCT05626569 |
|  | Short-course RT | Rectal cancer | 25 | II | Recruiting | NCT04109755 |
|  | Short-course RT | MSS locally advanced rectal adenocarcinoma | 32 | II | Active, not recruiting | NCT05216653 |
|  | IMRT | Stage III NSCLC | 8 | II | Active, not recruiting | NCT04577638 |
|  | RT | Urinary bladder cancer | 33 | II | Active, not recruiting | NCT03529890 |
|  | SBRT | Stage IV NSCLC | 5 | III | Active, not recruiting | NCT03867175 |
|  | RT | NSCLC | 37 | II | Active, not recruiting | NCT03523702 |
|  | RT | EC | 30 | I/II | Active, not recruiting | NCT03544736 |
|  | SBRT | NSCLC | 184 | II | Active, not recruiting | NCT03110978 |
|  | SBRT | Merkel cell carcinoma | 9 | II | Active, not recruiting | NCT03304639 |
|  | RT | HNC | 70 | II | Active, not recruiting | NCT03894891 |
|  | RT | UC | 13 | II | Active, not recruiting | NCT03486197 |
|  | RT | Breast cancer | 66 | I/II | Active, not recruiting | NCT03366844 |
|  | RT | HNSCC | 22 | I/II | Active, not recruiting | NCT03247712 |
|  | RT | Endometrial cancer | 168 | III | Active, not recruiting | NCT04214067 |
|  | HFRT | Recurrent rectal cancer | 75 | II | Not yet recruiting | NCT05628038 |
|  | SBRT | Locally advanced pancreatic cancer | 96 | II | Not yet recruiting | NCT06009029 |
|  | Postoperative RT | Locally advanced EC | 70 | I/II | Not yet recruiting | NCT05937438 |
|  | RT | Metastatic HNSCC | 290 | III | Not yet recruiting | NCT05721755 |
|  | RT | Breast cancer | 35 | II | Not yet recruiting | NCT05491226 |
|  | SBRT | NSCLC, STS, UC | 60 | NA | Not yet recruiting | NCT04587817 |
|  | SBRT | Locally advanced HNSCC | 23 | II | Not yet recruiting | NCT05861557 |
|  | RT | Urinary bladder cancer | 65 | II | Not yet recruiting | NCT05445648 |
|  | RT | Bladder cancer | 30 | II | Not yet recruiting | NCT05879653 |
|  | RT | ESCC | 30 | II | Not yet recruiting | NCT05791136 |
|  | RT | NPC | 28 | II | Not yet recruiting | NCT04917770 |
|  | Proton RT | Neoplasm metastasis | 30 | I/II | Unknown | NCT03765190 |
|  | SABR | Oligometastatic gastrointestinal cancer | 21 | I | Unknown | NCT04625894 |
|  | SABR | NSCLC | 30 | II | Unknown | NCT04271384 |
|  | SBRT | NSCLC | 31 | I/II | Unknown | NCT03383302 |
|  | SBRT | HCC | 116 | II/III | Unknown | NCT04167293 |
|  | SBRT | EC | 63 | II | Unknown | NCT04512417 |
|  | RT | NSCLC | 37 | II | Unknown | NCT04167657 |
|  | SABR | CRC | 60 | II | Unknown | NCT04535024 |
|  | RT | HCC | 39 | II | Unknown | NCT04193696 |
|  | RT | Thoracic malignancies | 45 | II | Unknown | NCT03732430 |
|  | SBRT | NSCLC | 20 | I/II | Unknown | NCT03446911 |
| Anti-PD-L1 | SBRT | Malignant mesothelioma | 15 | I/II | Completed | NCT03399552 |
|  | SABR | Breast cancer | 54 | II | Completed | NCT03464942 |
|  | RT | Oropharyngeal squamous cell carcinoma | 11 | II | Completed | NCT03623646 |
|  | RT | NSCLC | 7 | Early Phase I | Completed | NCT03818776 |
|  | HFRT | Glioblastoma | 6 | II | Completed | NCT02968940 |
|  | HFRT | Bladder cancer | 16 | II | Recruiting | NCT04543110 |
|  | HFRT | HNC | 18 | I | Recruiting | NCT04477759 |
|  | RT | Locally advanced thymic cancer | 25 | II | Recruiting | NCT06019468 |
|  | RT | SCLC | 138 | II/III | Recruiting | NCT04402788 |
|  | HFRT | Locally advanced NSCLC | 36 | I/II | Recruiting | NCT05269485 |
|  | RT | NSCLC | 90 | II | Recruiting | NCT04245514 |
|  | SRT | Advanced NSCLC | 52 | II | Recruiting | NCT04786093 |
|  | RT | Stage IV NSCLC | 20 | II | Recruiting | NCT04549428 |
|  | RT | Pancreatic cancer, virus-associated tumors, NSCLC, melanoma, bladder cancer, triple negative breast cancer | 247 | II | Recruiting | NCT03915678 |
|  | SBRT | Cervical cancer | 26 | II | Recruiting | NCT03614949 |
|  | RT | NSCLC | 10 | II | Recruiting | NCT05443971 |
|  | RT | NSCLC | 170 | III | Recruiting | NCT04597671 |
|  | RT | SCLC | 49 | II | Recruiting | NCT05068232 |
|  | RT | Rectal cancer | 38 | II | Recruiting | NCT04017455 |
|  | RT | Bladder cancer | 24 | II | Recruiting | NCT03747419 |
|  | EBRT | Metastatic merkel cell carcinoma | 38 | I/II | Recruiting | NCT04261855 |
|  | RT | Advanced penile cancer | 32 | II | Active, not recruiting | NCT03686332 |
|  | SBRT | HNSCC | 45 | I/II | Active, not recruiting | NCT03283605 |
|  | SBRT | Prostate cancer | 96 | II | Active, not recruiting | NCT03795207 |
|  | EBRT | Bladder cancer | 67 | II | Not yet recruiting | NCT03950362 |
|  | IMRT | HNC | 69 | I/II | Not yet recruiting | NCT03051906 |
|  | SRS | Brain metastases from NSCLC | 46 | II | Not yet recruiting | NCT04889066 |
|  | SBRT | NSCLC | 106 | II | Unknown | NCT03446547 |
| Anti-CTLA-4 | IMRT | NSCLC | 39 | I/II | Completed | NCT02221739 |
|  | SBRT | Liver cancer, lung cancer | 143 | I | Completed | NCT02239900 |
| Anti-PD-1, anti-PD-L1 | HFRT | NSCLC | 35 | NA | Completed | NCT03035890 |
|  | SBRT | NSCLC | 3 | II | Completed | NCT03825510 |
|  | SRS | Brain metastases from melanoma or NSCLC | 190 | III | Recruiting | NCT05522660 |
|  | SRT | Locally advanced NSCLC | 112 | III | Recruiting | NCT05111197 |
|  | HFRT | Advanced NSCLC | 40 | I/II | Recruiting | NCT05754203 |
|  | RT | HNSCC, NSCLC | 200 | NA | Recruiting | NCT04892849 |
|  | Short-course RT | HNSCC, NSCLC | 57 | II | Recruiting | NCT03313804 |
|  | Radical-dose image guided RT | Stage IV NSCLC | 44 | II | Active, not recruiting | NCT03176173 |
|  | SBRT | Metastatic cancer | 146 | II | Active, not recruiting | NCT02843165 |
| Anti-PD-1, anti-CTLA-4 | SBRT | RCC | 29 | II | Completed | NCT03065179 |
|  | EBRT | Melanoma | 20 | I | Completed | NCT02659540 |
|  | RT | Metastatic CRC | 19 | I | Completed | NCT03507699 |
|  | SBRT | Stage IV NSCLC | 427 | II/III | Recruiting | NCT04929041 |
|  | RT | CRC, pancreatic cancer | 80 | II | Recruiting | NCT03104439 |
|  | SRS | Melanoma brain metastases | 10 | I | Recruiting | NCT05341349 |
|  | SBRT | Melanoma with liver metastases | 18 | I | Recruiting | NCT05169957 |
|  | SRS | Stage IV melanoma | 218 | II | Recruiting | NCT03340129 |
|  | SBRT | Metastatic RCC | 78 | II | Recruiting | NCT04090710 |
|  | RT | STS | 14 | Early Phase I | Active, not recruiting | NCT03463408 |
|  | RT | Gliosarcoma | 485 | II/III | Active, not recruiting | NCT04396860 |
|  | SBRT | Stage IV SCLC | 78 | I | Active, not recruiting | NCT03223155 |
|  | SRS | Brain metastases | 200 | NA | Active, not recruiting | NCT03458455 |
|  | RT | Pancreatic cancer | 30 | II | Active, not recruiting | NCT04361162 |
|  | RT | SCLC | 21 | I/II | Active, not recruiting | NCT03043599 |
|  | RT | Uveal melanoma, hepatic metastases | 26 | I/II | Unknown | NCT02913417 |
| Anti-PD-L1, anti-CTLA-4 | SBRT | Liver metastases from CRC | 22 | II | Completed | NCT03101475 |
|  | SBRT | Metastatic anaplastic thyroid cancer | 13 | I | Completed | NCT03122496 |
|  | LDRT | NSCLC | 29 | II | Recruiting | NCT05000710 |
|  | EBRT | Invasive bladder cancer | 32 | II | Active, not recruiting | NCT03702179 |
|  | RT | STS | 22 | I/II | Active, not recruiting | NCT03116529 |
|  | SBRT | Oropharyngeal squamous cell cancer | 82 | I/II | Active, not recruiting | NCT03618134 |
| Anti-PD-L1, anti-TIGIT | SBRT | Metastatic NSCLC, metastatic bladder cancer, metastatic RCC, metastatic HNC | 92 | I | Not yet recruiting | NCT05259319 |
| Anti-PD-1, anti-LAG-3 | SBRT | Uveal melanoma | 40 | II | Recruiting | NCT05077280 |
| Anti-PD-1, anti-4-1BB | SBRT | Multiple metastases in advanced solid tumors | 60 | I | Completed | NCT03431948 |
| Anti-PD-1, BO-112 | SABR | Metastatic NSCLC | 30 | I/II | Recruiting | NCT05265650 |
| Anti-PD-1, anti-PD-L1, anti-CTLA-4 | SBRT | Melanoma, RCC, NSCLC, bladder cancer | 60 | II | Recruiting | NCT03693014 |
| ICB | SBRT | Pleural mesothelial neoplasm | 20 | I | Recruiting | NCT04926948 |
|  | SRS | NSCLC, RCC, breast carcinoma, melanoma, brain metastases | 244 | NA | Recruiting | NCT05703269 |
|  | SBRT | HCC | 33 | II | Active, not recruiting | NCT03817736 |
| Anti-OX40 | SBRT | Metastatic breast cancer | 14 | I | Completed | NCT01862900 |
| Anti-CD20 | RT | Follicular lymphoma | 190 | III | Not yet recruiting | NCT05929222 |
| L19-IL2 | SABR | Metastatic NSCLC | 126 | II | Recruiting | NCT03705403 |
| NHS-IL12 | SBRT | Prostate cancer | 65 | II | Recruiting | NCT05361798 |
| Anti-PD-1, CD80/IL2 | RT | Advanced solid tumor | 374 | I/II | Recruiting | NCT04977453 |
| IL-2, anti-PD-1, anti-CTLA-4 | RT | Metastatic melanoma | 4 | II | Completed | NCT03850691 |
| FLT3 ligand immunotherapy | SBRT | NSCLC | 29 | II | Completed | NCT02839265 |
| Cancer vaccine | RT | Liver cancer | 12 | I | Completed | NCT00081848 |
|  | RT | Prostate cancer | 48 | II | Completed | NCT00005916 |
|  | RT | Lung cancer | - | II | Completed | NCT00006470 |
| Oncolytic virus | RT | Malignant gliomas | 15 | I | Completed | NCT00751270 |
|  | LDRT | Cerebellar brain tumor | 15 | I | Recruiting | NCT03911388 |
|  | RT | Prostate cancer | 711 | III | Active, not recruiting | NCT01436968 |
|  | LDRT | Supratentorial brain tumors | 13 | I | Active, not recruiting | NCT02457845 |
|  | LDRT | High-grade glioma | 40 | II | Not yet recruiting | NCT04482933 |
| Anti-PD-1, cancer vaccine | SBRT | Pancreatic cancer | 30 | II | Active, not recruiting | NCT03161379 |
| Anti-PD-1, anti-CTLA-4, cancer vaccine | SBRT | Pancreatic cancer | 20 | I | Recruiting | NCT05721846 |
| Anti-PD-1, anti-CTLA-4, influenza vaccine | SBRT | Pancreatic cancer | 30 | II | Recruiting | NCT05116917 |
| GM-CSF, cancer vaccine, anti-CD3, IL-2 | RT | Astrocytoma or oligodendroglioma | 60 | II | Completed | NCT00004024 |
| Autologuos lymphoid effector cells specific against tumor (ALECSAT) | RT | Glioblastoma | 62 | II | Completed | NCT02799238 |
| T regulatory and T conventional cell adoptive immunotherapy | Fractionated total body irradiation or total marrow and lymph node irradiation | Acute myeloid leukemia, acute lymphoid leukemia, lymphoma, multiple myeloma | 80 | II | Unknown | NCT03977103 |
| Dendritic cell injections | EBRT | STS | 17 | II | Completed | NCT00365872 |
| Adoptive cellular therapy | IMRT | EC | 40 | NA | Unknown | NCT01691664 |
| Anti-CD40, poly ICLC, recombinant FLT3 ligand | RT | Breast cancer, melanoma | 18 | I | Recruiting | NCT04616248 |

RT: radiotherapy; PD-1: programmed cell death 1; PD-L1: programmed cell death ligand 1; CTLA-4: cytotoxic T lymphocyte-associated protein 4; TIGIT: T cell immunoreceptor with Ig and ITIM domains; LAG-3: lymphocyte activation gene 3; ICB: immune checkpoint blockade; HDCRT: high-dose conformal radiation therapy; HDRT: high dose radiotherapy; SBRT: stereotactic body radiotherapy; SRT: stereotactic radiation therapy; IMRT: intensity modulated-radiotherapy; LDRT: low dose radiotherapy; EBRT: external beam radiation therapy; CIRT: carbon ion radiotherapy; SRS: stereotactic radiosurgery; HFRT: hypofractionated radiotherapy; SABR: stereotactic ablative radiotherapy; NSCLC: non-small-cell lung cancer; UC: urothelial cancer; HNC: head and neck cancer; HNSCC: head and neck squamous cell carcinoma; NPC: nasopharyngeal carcinoma; STS: soft tissue sarcoma; ESCC: esophageal squamous cell carcinoma; EC: esophageal carcinoma; HCC: hepatocellular carcinoma; CRC: colorectal cancer; SCLC: small-cell lung cancer; RCC: renal cell carcinoma
